# MRI-based classification of IDH mutation and 1p/19q codeletion status of gliomas using a 2.5D hybrid multi-task convolutional neural network

**DOI:** 10.1093/noajnl/vdad023

**Published:** 2023-03-05

**Authors:** Satrajit Chakrabarty, Pamela LaMontagne, Joshua Shimony, Daniel S Marcus, Aristeidis Sotiras

**Affiliations:** Department of Electrical and Systems Engineering, Washington University in St. Louis, St. Louis, MO, USA; Mallinckrodt Institute of Radiology, Washington University School of Medicine, St. Louis, MO, USA; Mallinckrodt Institute of Radiology, Washington University School of Medicine, St. Louis, MO, USA; Mallinckrodt Institute of Radiology, Washington University School of Medicine, St. Louis, MO, USA; Mallinckrodt Institute of Radiology and Institute for Informatics, Washington University School of Medicine, St. Louis, USA

**Keywords:** deep learning, glioma, isocitrate dehydrogenase, overall survival, 1p/19q codeletion

## Abstract

**Background:**

IDH mutation and 1p/19q codeletion status are important prognostic markers for glioma that are currently determined using invasive procedures. Our goal was to develop artificial intelligence-based methods to noninvasively determine molecular alterations from MRI.

**Methods:**

Pre-operative MRI scans of 2648 glioma patients were collected from Washington University School of Medicine (WUSM; *n* = 835) and publicly available Brain Tumor Segmentation (BraTS; *n* = 378), LGG 1p/19q (*n* = 159), Ivy Glioblastoma Atlas Project (Ivy GAP; *n* = 41), The Cancer Genome Atlas (TCGA; *n* = 461), and the Erasmus Glioma Database (EGD; *n* = 774) datasets. A 2.5D hybrid convolutional neural network was proposed to simultaneously localize glioma and classify its molecular status by leveraging MRI imaging features and prior knowledge features from clinical records and tumor location. The models were trained on 223 and 348 cases for IDH and 1p/19q tasks, respectively, and tested on one internal (TCGA) and two external (WUSM and EGD) test sets.

**Results:**

For IDH, the best-performing model achieved areas under the receiver operating characteristic (AUROC) of 0.925, 0.874, 0.933 and areas under the precision-recall curves (AUPRC) of 0.899, 0.702, 0.853 on the internal, WUSM, and EGD test sets, respectively. For 1p/19q, the best model achieved AUROCs of 0.782, 0.754, 0.842, and AUPRCs of 0.588, 0.713, 0.782, on those three data-splits, respectively.

**Conclusions:**

The high accuracy of the model on unseen data showcases its generalization capabilities and suggests its potential to perform “virtual biopsy” for tailoring treatment planning and overall clinical management of gliomas.

**Key Points**

1. Hybrid 2.5D model jointly detects, segments glioma, and classifies molecular subtypes from MRI.
2. Combining clinical knowledge with imaging features outperforms conventional convolutional neural network.
3. External validation on 968 patients from 11 centers shows good model generalization.

**Importance of the Study**

We propose a 2.5D multi-task hybrid convolutional neural network for classifying IDH mutation and 1p/19q codeletion status of gliomas of all grades. Our model jointly detects and segments glioma before classifying its molecular status, thus obviating any requirement of multiple task-specific models. Additionally, the model integrates prior clinical knowledge through a feature-fusion mechanism. Aggregating information from three orthogonal planes provides the model with richer spatial context than a 2D model without incurring the computational burden of a 3D model. To facilitate clinical translation, no patient cases were excluded based on image acquisition parameters, image quality, or glioma grade. Extensive validation of the model on three independent hold-out sets comprising 968 patient cases from 11 different sites demonstrated good generalization. Head-to-head comparisons were performed to two baseline methods to explore the methodological, computational and performance advantages of the model. The code and trained models of this work are available.

Gliomas are characterized by distinct imaging characteristics, response to therapy, prognoses, and varying survival rate. As per the World Health Organization (WHO) guidelines,^1,2^ the definition of these tumors requires integrating histological information with molecular parameters. Two of the most important molecular markers are the mutation status of isocitrate dehydrogenase (IDH) enzyme and the codeletion of chromosome arms 1p and 19q (1p/19q). These markers have unique prognostic significance that can considerably impact treatment planning. Therefore, their accurate determination can significantly improve patient outcome.

In clinical settings, gliomas are routinely resected at first appearance considering their potentially grim prognosis. The tissue-sample obtained from resection or biopsy procedures are used to determine IDH and 1p/19q status using immunohistochemistry (IHC). However, this can have associated risk,^3^ may fail to capture intra-tumoral spatial heterogeneity, can be inaccessible in low-resource settings, or can lack adequate tumor content or optimal quality and quantity of nucleic acid required for correct molecular characterization.^4^ Therefore, noninvasive imaging techniques, like MRI, have been investigated as complementary “virtual biopsy” procedures that can be potentially used to determine the molecular status of the gliomas even before the first resection, thus facilitating easier clinical decision-making.

Artificial intelligence-based approaches^5^ have attempted to perform molecular assessment by leveraging the variation in tumor phenotypical characteristics manifested in MRI scans due to changes in molecular alterations.^6–10^ Several studies^11,12^ have investigated machine learning (ML) approaches in conjunction with radiomic features for this purpose. However, these methods are limited by their requirement of separately generated tumor mask, manual feature selection, and reproducibility issues associated with radiomic features.^13^ On the other hand, deep learning (DL) approaches^5^ overcome these limitations by automatically learning hierarchical imaging features. Nevertheless, several challenges still limit their adoption in routine clinical practice. First, similar to ML methods, most existing DL methods require a manually drawn,^14–16^ or automatically generated^6,17–19^ tumor segmentation mask. Manual delineation of tumor masks is tedious and prone to human error and observer bias, whereas automatically generated masks require an additional task-specific model. Such task-specific models not only increase computational burden but also fail to leverage the context between different related tasks. To address this, multi-task DL models have been proposed.^18,20^ However, these focus solely on imaging information and fail to incorporate prior clinical knowledge. Second, most studies have assessed their methods either on one type of molecular status (only IDH mutation^15,21^ or 1p/19q codeletion^10^) or specific grades of glioma (eg, only low grade^14,17^). This failed to provide a comprehensive classification system that aligns with WHO classification and recognizes the importance of combined IDH and 1p/19q status prediction. Third, previous studies often used small samples and lacked rigorous external validation,^14,16^ which is necessary for accurately assessing model generalizability. Fourth, existing studies have used varying datasets and performance metrics that make objective comparisons between various methods challenging. Without head-to-head comparisons and data-driven conclusions, it is difficult to gauge the advancements in the field and identify the best-performing methods.

To address these limitations, we propose a 2.5D multi-task hybrid convolutional neural network (CNN) approach for classifying both IDH mutation and 1p/19q codeletion status of high- and low-grade gliomas (grades 2–4) from routine MR sequences (ie, pre-operative postcontrast T1-weighted (T1c), T2-weighted (T2), and T2-weighted Fluid-attenuated inversion recovery (FLAIR). Our model jointly detects and segments the glioma before classifying its molecular status, thus obviating any additional tumor segmentation step. Additionally, it can integrate prior knowledge through a feature-fusion mechanism. We train the model on three orthogonal planes viz. axial, coronal, and sagittal, thus providing the model with richer spatial context compared to 2D models without incurring the computational burden of a 3D model. We assembled the largest sample till date for a study of this kind, consisting of 2648 patients from 14 institutions. The model has been extensively validated on 3 independent hold-out sets comprising 968 patient cases from 11 different institutions, to demonstrate its generalizability.

## Materials and Methods

### Datasets

**Figure 1.**
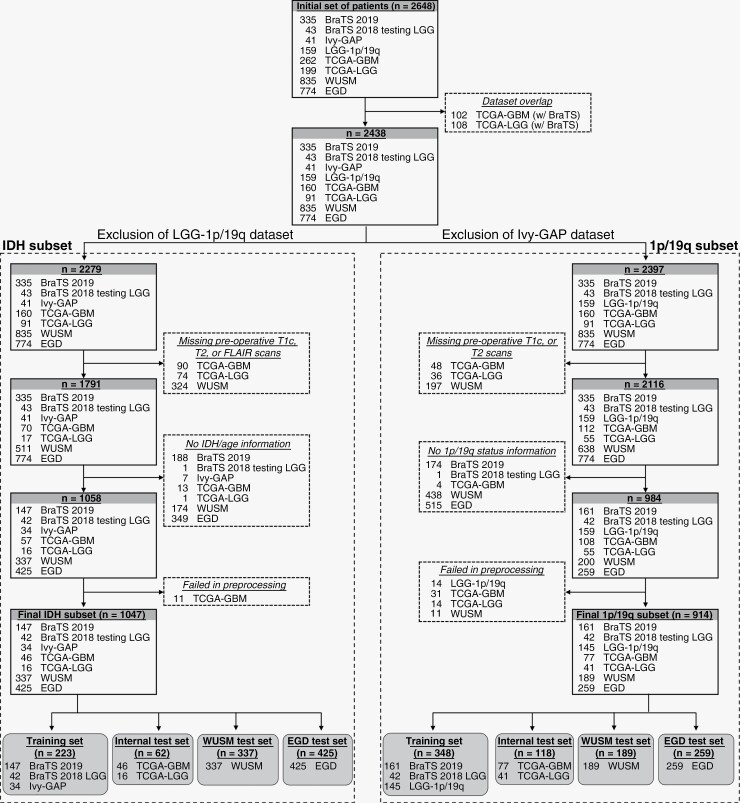
Inclusion flowchart of data for IDH mutation and 1p/19q codeletion experiments. The underlined and italicized text within each box with dashed lines details the reason for exclusions. BraTS: Brain Tumor Segmentation dataset, Ivy-GAP: Ivy Glioblastoma Atlas Project, TCGA-GBM: The Cancer Genome Atlas Glioblastoma Multiforme, TCGA-LGG: The Cancer Genome Atlas Low Grade Glioma, WUSM: Washington University School of Medicine, EGD: Erasmus Glioma Database, T1c: postcontrast T1-weighted scan, T2: T2-weighted scan, FLAIR: Fluid Attenutated Inversion Recovery scan.

Retrospective pre-operative MRI scans from 2648 patients with gliomas CNS WHO grades 2-4, confirmed using an integrated histopathological and molecular definition, were considered for inclusion in the study (Figure 1). Data were acquired from 7 publicly available datasets across 13 different institutions: Brain Tumor Segmentation^22–24^ (BraTS; *n* = 378), LGG 1p/19q^25^ (*n* = 159), Ivy Glioblastoma Atlas Project^26^ (Ivy GAP; *n* = 41), The Cancer Genome Atlas^27,28^ (TCGA; *n* = 461), and the Erasmus Glioma Database^29^ (EGD; *n* = 774). Additional data were acquired from retrospective health records of Washington University School of Medicine (WUSM; *n* = 835). Overlapping cases between the TCGA and BraTS data collections were removed. No patient cases were excluded based on image acquisition parameters or image quality to mirror the inherent heterogeneity present in clinical data. Additionally, to ensure a wide applicability of the method, only routine MRI sequences were used, and no exclusions were made based on glioma grade.

Based on study requirements, two different but overlapping subsets of data were considered for IDH and 1p/19q status classification. For both classification tasks, the training sets included only cases with available expert tumor segmentations (Supplementary Data S1.1), required to train the model. An internal test set, and two additional external sets were included for each task to accurately estimate model generalizability.

For compilation of the IDH database, the inclusion criteria were: (1) pathologically confirmed glioma CNS WHO grades 2-4, (2) known IDH status (Supplementary Data S1.1) and patient age at diagnosis, and (3) presence of T1c, T2, and FLAIR scans. Based on these criteria, 1047 patient cases were selected. These were subsequently split into four sets: cross-validation (*n* = 223 from BraTS and Ivy GAP), internal testing (*n* = 62 from TCGA), and two external test sets viz. WUSM (*n* = 337) and EGD (*n* = 425).

For compilation of the 1p/19q database, the following inclusion criteria were considered: (1) pathologically confirmed glioma CNS WHO grades 2-4, (2) known 1p/19q codeletion status (Supplementary Data S1.1), and (3) presence of T1c and T2 scans. We did not require the presence of FLAIR because the LGG 1p/19q dataset comprised only T1c and T2 scans. Based on these criteria, 914 patient cases were selected. These were subsequently split into four sets: cross-validation (*n* = 348 from BraTS and LGG 1p/19q), internal testing (*n* = 118 from TCGA), and two external test sets viz. WUSM (*n* = 189) and EGD (*n* = 259).

### Image Acquisition, Preprocessing, and Feature Extraction

Due to being acquired from 8 different sources across 14 different institutions, the data were extremely heterogeneous exhibiting high variability in acquisition protocol parameters (Supplementary Data S1.2, Supplementary Figures S1-S5). Data were either already preprocessed following the BraTS pre-processing protocol^30^ during collection or pre-processed during the study using the Integrative Imaging Informatics for Cancer Research: Workflow Automation for Neuro-oncology (I3CR-WANO) framework^31^ (Supplementary Data S1.3, S2.7).

Besides imaging data, two prior knowledge features viz. patient age at diagnosis (hereon referred to as “age”) and anatomical location of tumor (hereon referred to as “loc”) were included in the network (Supplementary Data S1.3).

### Hybrid 2.5D Multi-task Model Architecture

**Figure 2.**
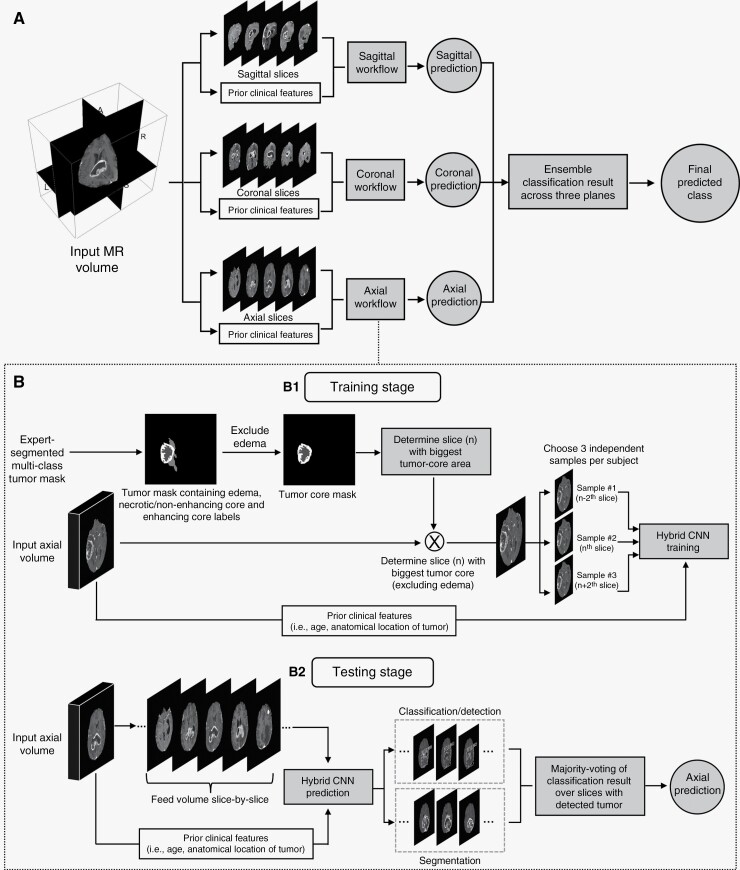
Detailed schematic of the proposed deep learning system for predicting molecular status. (A) Multi-view aggregation architecture that combines predictions from planar models trained independently on 2D slices along three principal axes (axial, coronal, sagittal). The final classification result is obtained by aggregating the classification result from all three networks. (B) Each planar 2D network is based on a hybrid network that simultaneously detects and segments the tumor and classifies the molecular status, based on 2D slices and prior knowledge features. During training (subpanel B1) expert-segmented multi-class tumor masks are used to (i) select 2D slices and (ii) supervise the tumor detection and segmentation tasks. During testing (subpanel B2), the 2D planar model is applied to all available slices and a prediction is made through consensus of predictions for slices where a tumor was detected. For ease of visualization, we show only the axial workflow for one modality (postcontrast T1 weighted), but models work across three planes for multiple modalities. MR: Magnetic Resonance, CNN: Convolutional Neural Network.

We adopted a 2.5D approach, aiming to capture richer spatial context compared to 2D models, while minimizing computational requirements. Specifically, we train a separate 2D model for each orthogonal plane (ie, axial, coronal, sagittal), whose predictions are combined into the final result through a multi-view aggregation step (Figure 2).

The end goal of our model is to classify the molecular status. However, due to the sparse presence of glioma in the MRI image, the classification performance of the network might get affected by nontumorous image characteristics. To resolve this, the proposed 2D models follow a Mask RCNN architecture^32^ and tackle two auxiliary tasks of glioma detection and segmentation besides the classification task (Supplementary Data S1.4, Supplementary Figure S6). Additionally, we augment the 2D models into a hybrid architecture, which integrates imaging features with prior knowledge features.

CNNs are mostly image-intensity based, and hence are unable to take demographic features (eg, patient age) or neuroanatomical features (eg, tumor location features) into account. This is limiting given ample evidence regarding the importance of patient age in predicting IDH status^33^ and the association between tumor location and 1p/19q codeletion status.^6,9,10^ To address this, we equipped our CNN with a late-fusion strategy^34^ to exploit additional features (ie, “age” and “loc” features), thus combining the strengths of image-derived features with clinical prior knowledge (Supplementary Data S1.4, Supplementary Figure S6A). Subsequently, this set of hybrid features is passed through a fully connected layer to the final classification layer of the network. The training and testing processes involving the hybrid features are end-to-end (Figure 2, Supplementary Data S1.5). The code and trained models of this work are available at https://github.com/satrajitgithub/glioma_molecular_2.5D.git.

### Statistical Analysis

We used Chi-square and Mann–Whitney tests to evaluate differences in patient demographics and clinical characteristics between data splits. We performed ablation studies to determine the importance of prior knowledge features (Supplementary Data S1.6). Additionally, we investigated the effectiveness of aggregating information from three planes (ie, the 2.5D approach) compared to 2D (Supplementary Data S1.6) and 3D models (Supplementary Data S2.5). The performance of the best-performing model was compared to two baseline pre-trained models: (1) a multi-task U-net model by Voort et al.^20^ (hereon “Voort-CNN”) for both IDH and 1p/19q, and (2) a CNN-radiomics hybrid model by Choi et al.^19^ (hereon “Choi-CNN”) for only IDH (Supplementary Data S1.7). Also, considering the randomness involved in DL approaches resulting in nondeterministic results, we have repeated the experiments with the best performing models of both IDH and 1p/19q five times in addition to the reported results and compared the performance across all runs to determine any differences (Supplementary data S1.10, S2.8).

The classification performance was quantified using accuracy, precision, recall, F1 score, area under receiver operating characteristics (AUROC), and area under precision-recall curves (AUPRC). For AUROC and AUPRC, 95% confidence intervals (CI) were calculated using a 1000-sample bootstrapping method (Supplementary data S1.8). Confusion matrices were calculated to show the error distribution across different classes. Statistical comparisons between methods were performed using the McNemar test^35^ for precision, the generalized score statistic^36^ for recall, and the DeLong test^37^ for AUROCs.

To assess the validity of our model in terms of WHO 2016 and WHO 2021 glioma subtypes, we compared OS based on ground truth vs. predicted molecular status (Supplementary Data S1.9). We hypothesized that the misclassified IDH-wt cases with IDH-mut like phenotype will have better OS. Accordingly, we used Kaplan–Meier survival curves to characterize and compare groups of misclassified cases (ie, IDH-wt predicted as IDH-mut and vice versa) in WUSM in terms of OS. Differences in the Kaplan–Meier curves were analyzed using Cox regression. Additionally, we examined recurring patterns in misclassified cases for both IDH and 1p/19q classification tasks (Supplementary data S1.9).

### Ethics Statement

Retrospective de-identified data were obtained from WUSM, with a waiver of consent in accordance with the Health Insurance Portability and Accountability Act, as approved by the Institutional Review Board (IRB ID # 202004209). Additional data were obtained from public datasets after completion of necessary data usage agreements.

## Results

### Dataset Characteristics

Patient demographics and clinical characteristics were calculated for all datasets created for both prediction tasks (Table 1). For both IDH and 1p/19q subsets, the internal, WUSM, and EGD sets differed to varying degrees in terms of age, sex, and clinical characteristics (Supplementary Data S2.1).

**Table 1.** Patient characteristics stratified by train, test, WUSM, and EGD splits of data

				IDH							1p/19q			
	Train (*n* = 223)	Test (*n* = 62)	P-value (train vs.test)	WUSM (*n* = 337)	P-value (train vs.WUSM)	EGD (*n* = 425)	P-value (train vs. EGD)	Train (*n* = 348)	Test (*n* = 117)	P-value (train vs. test)	WUSM (*n* = 189)	P-value (train vs. WUSM)	EGD (*n* = 248)	P-value (train vs. EGD)
Age	54 (41 62)	58 (49– 66)	0.041	59 (46–68)	<0.001	57 (45–69)	0.0017	48 (35–59)	56 (38–66)	0.001	48 (33–62)	0.8282	48 (37–58)	0.8613
Sex			0.4838		0.0406		0.0265			0.9323		0.004		0.0107
Female	107 (47.98%)	26 (41.94%)		131 (38.87%)		164 (38.59%)		164 (47.13%)	55 (47.01%)		64 (33.86%)		90 (36.29%)	
Male	116 (52.02%)	36 (58.06%)		206 (61.13%)		261 (61.41%)		184 (52.87%)	62 (52.99%)		125 (66.14%)		158 (63.71%)	
IDH status			0.2829		<0.001		0.1757							
Mutant	91 (40.81%)	20 (32.26%)		88 (26.11%)		149 (35.06%)		–	–		–		–	
Wild-type	132 (59.19%)	42 (67.74%)		249 (73.89%)		276 (64.94%)		–	–		–		–	
1p/19q codeletion status										<0.001		0.9945		0.1356
Codeleted	–	–		–		–		121 (34.77%)	16 (13.68%)		65 (34.39%)		71 (28.63%)	
Noncodeleted	–	–		–		–		227 (65.23%)	101 (86.32%)		124 (65.61%)		177 (71.37%)	
CNS WHO Grade			<0.001		<0.001		<0.001			<0.001		<0.001		<0.001
2	47 (21.08%)	11 (17.74%)		66 (19.58%)		124 (29.18%)		143 (41.09%)	28 (23.93%)		91 (48.15%)		126 (50.81%)	
3	59 (26.46%)	5 (8.06%)		47 (13.95%)		29 (6.82%)		109 (31.32%)	13 (11.11%)		26 (13.76%)		39 (15.73%)	
4	104 (46.64%)	46 (74.19%)		222 (65.88%)		256 (60.24%)		96 (27.59%)	76 (64.96%)		72 (38.1%)		65 (26.21%)	
Unknown	13 (5.83%)			2 (0.59%)		16 (3.76%)							18 (7.26%)	

Percentage values in parentheses represent the proportion of each subgroup within a specific dataset (ie, train, test, WUSM, EGD). For “Age” row, interquartile range (IQR) is given in parentheses.

Abbreviations: WUSM, Washington University School of Medicine; EGD, Erasmus Glioma Database.

### IDH Mutation Status Prediction

#### Classification performance

**Figure 3.**
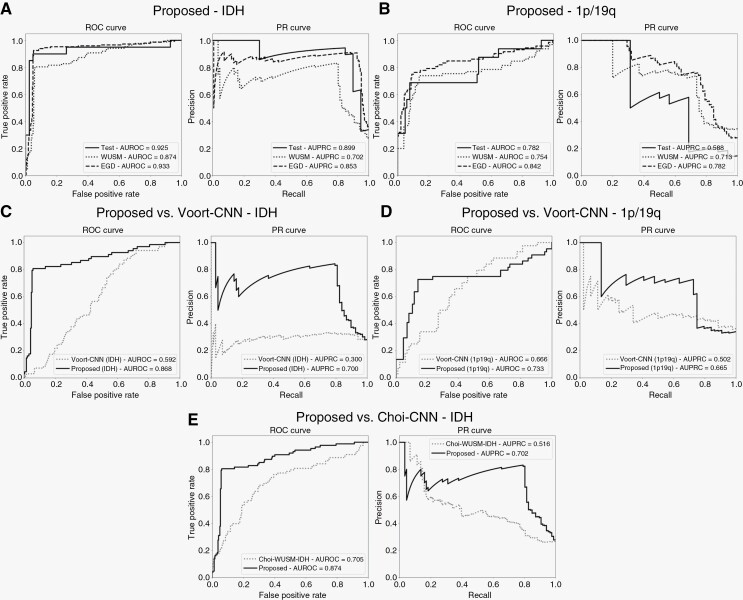
The proposed model achieved better classification performance in both IDH status (P < 0.05 for precision, recall and AUROC) and 1p/19q status (*P* < 0.05 for recall) prediction tasks than the baseline methods. ROC and PR curves are reported for (A) the proposed model (CNN+age) for IDH status prediction; (B) the proposed model (CNN+loc) for 1p/19q status prediction; (C) the comparison of the proposed model performance to Voort-CNN^20^ for IDH prediction; (D) the comparison of the proposed model performance to Voort-CNN^20^ for 1p/19q prediction; and (E) the comparison of the proposed model performance to Choi-CNN^19^ for IDH prediction. ROC: Receiver Operating Characteristics, PR: Precision-Recall.

Our ablation studies (Supplementary Data S2.2) determined the 2.5D CNN+age model to be the best-performing configuration for IDH status classification (Table 2, Figure 3A, Supplementary Figure S7A). This model yielded high accuracies on the internal (93.5%), WUSM (90.4%), and EGD (94.1%) test sets. Compared to the internal test set, the model exhibited a 0.11 drop in precision (0.831), and very minor drops in recall (0.793) and AUROC values (0.874, 95% CI: 0.826–0.917) on the WUSM test set. For the EGD set, it yielded a similar precision (0.908), and minor improvements in recall (0.926) and AUROC (0.933, 95% CI: 0.0.902–0.960) compared to the internal test set. Overall, the model showed good generalization on both external sets, with the performance being slightly better in EGD compared to WUSM.

**Table 2.** Performance of proposed model and comparison with Voort-CNN and Choi-CNN for prediction of IDH mutation and 1p/19q codeletion status

	Accuracy	Precision	Recall	F1-score	AUROC	AUROC-95% CI	AUPRC	AUPRC-95% CI
IDH								
Test	0.935	0.944	0.850	0.895	0.925	(0.809, 1.000)	0.899	(0.740, 1.000)
WUSM	0.904	0.831	0.793	0.812	0.874	(0.826, 0.917)	0.702	(0.600, 0.812)
EGD	0.941	0.908	0.926	0.917	0.933	(0.902, 0.960)	0.853	(0.780, 0.918)
1p/19q								
Test	0.881	0.579	0.687	0.628	0.782	(0.627, 0.916)	0.588	(0.354, 0.810)
WUSM	0.819	0.738	0.738	0.738	0.754	(0.666, 0.840)	0.713	(0.611, 0.813)
EGD	0.853	0.724	0.764	0.743	0.842	(0.776, 0.904)	0.782	(0.697, 0.860)
Comparison with Voort								
Voort-CNN (IDH)	0.701	0.449	0.580	0.506	0.592	(0.522, 0.668)	0.300	(0.237, 0.402)
Proposed (IDH)	0.907	0.844	0.794	0.818	0.868	(0.810, 0.921)	0.700	(0.585, 0.826)
Voort-CNN (1p/19q)	0.667	0.667	0.045	0.085	0.666	(0.568, 0.759)	0.502	(0.377, 0.654)
Proposed (1p/19q)	0.814	0.727	0.727	0.727	0.733	(0.630, 0.834)	0.665	(0.539, 0.807)
Comparison with Choi								
Choi-CNN (IDH)	0.691	0.438	0.648	0.523	0.705	(0.636, 0.767)	0.516	(0.418, 0.612)

Abbreviations: IDH, isocitrate dehydrogenase; WUSM, Washington University School of Medicine; EGD, Erasmus Glioma Database; AUROC, area under the receiver operating characteristic curve; AUPRC, area under the precision-recall curve; CI, confidence interval.

Compared to the Voort-CNN baseline, this model performed significantly better with a higher precision (0.391 increase; *P* < 0.001), recall (0.22 increase; *P* = 0.004), and AUROC (0.113 increase; *P* = 0.002) (Figure 3C, Supplementary Table S1, Supplementary Figure S7C). Similar improvements were obtained compared to the Choi-CNN baseline (Figure 3E, Supplementary Table S1, Supplementary Figure S7E) in terms of precision (0.393 increase; *P* < 0.001), recall (0.149 increase; *P* = 0.028), and AUROC (0.17 increase; *P* < 0.001).

### Failure Analysis and Correlation with Overall Survival

We identified the following main sources of error. First, given that the classification predictions are contingent upon successful tumor detections, we observed that the model failed to make any molecular status classification due to undetected tumors for a small number of cases (3 of 337 and 1 of 425 cases in WUSM and EGD sets, respectively; columns marked with “BG” or background, Supplementary Figure S7A). Second, classifications were sometimes affected by poor off-plane resolution, specifically in the EGD test set (4 of the 11 IDH-mut cases misclassified as IDH-wt) (Supplementary Data S2.3).

**Figure 4.**
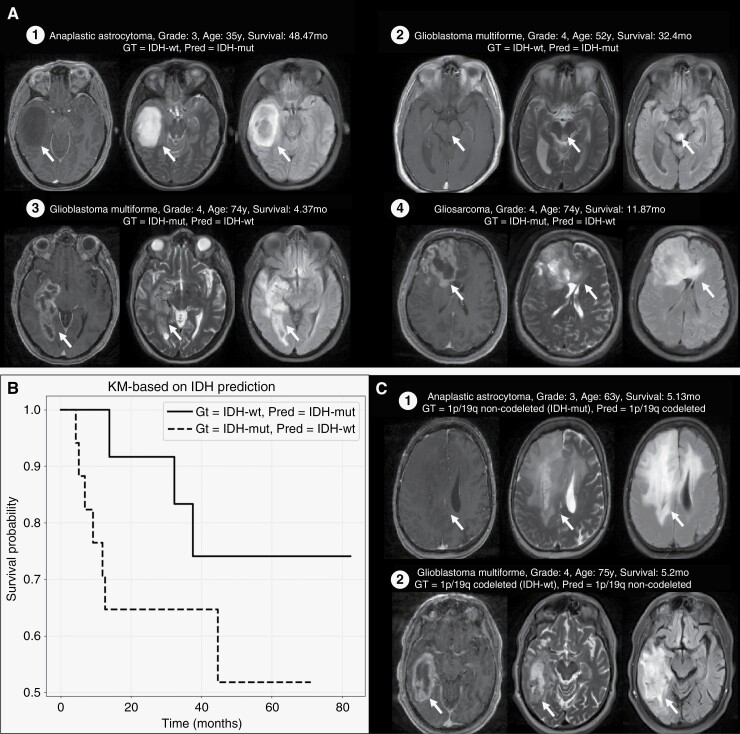
Case study of model misclassifications in IDH and 1p/19q prediction tasks. (A) T1c, T2 and FLAIR axial slices of four exemplary tumor cases which were misclassified in IDH prediction, (B) Kaplan–Meier survival curves characterizing the overall survival for predicted IDH-mut and predicted IDH-wt groups from misclassified cases of WUSM test set in the IDH prediction task, and (C) T1c, T2, and FLAIR axial slices of two exemplary tumor cases which were misclassified in 1p/19q prediction.

Comparison of OS demonstrated that there was a high alignment between the OS of patients based on ground truth WHO 2016 and WHO 2021 subtypes and corresponding predicted subtypes (Supplementary Figure S8). Analysis of the misclassifications showed that for most of the misclassified cases, the predicted IDH status had a better concordance than the IDH ground-truth label with tumor phenotype, patient age at diagnosis, and OS (Figure 4A, Supplementary Data S2.3). Overall, the group predicted as IDH-mut had a higher median OS than the one predicted as IDH-wt (47.6 vs.16.94 months) (Figure 4B).

### 1p/19q Codeletion Status Prediction

#### Classification performance

Our ablation studies (Supplementary Data S2.4) determined the 2.5D CNN+loc model to be the best-performing configuration for 1p/19q codeletion status classification (Table 2, Figure 3B, Supplementary Figure S7B). This model achieved high accuracies on the internal (88.1%) and EGD (85.3%) test sets, with a minor drop on the WUSM (81.9%) set. Precision and recall metrics in the internal test set were affected by a small percentage of false-positive cases (7.8%, 8 of 102) due to the high class imbalance therein (13.5% codeleted vs. 86.5% noncodeleted). Compared to the internal test set (57.9%), the precision was much higher on the WUSM (73.8%) and EGD (72.4%) sets. Similarly, model recall was much higher on the WUSM (73.8%) and EGD (76.4%) sets compared to the internal test set (62.5%). This led to a much higher AUPRC for the WUSM (0.713, 95% CI: 0.611–0.813) and EGD (0.782, 95% CI: 0.697–0.860) sets compared to the internal (0.588, 95% CI: 0.354–0.810) test set. However, the model achieved similar AUROC values for all three datasets (0.782, 95% CI: 0.627–0.916 for the internal test set; 0.754, 95% CI: 0.666–0.840 for the WUSM test set; and 0.842, 95% CI: 0.776–0.904 for the EGD test set). This disparity in AUROC and AUPRC can be explained by the severe class-imbalance in the internal test data (Supplementary Data S2.6). Overall, the model performed better on both external test sets compared to the internal test set, with the performance in EGD being slightly better than WUSM.

Compared to the Voort-CNN (Figure 3D, Supplementary Table S2, Supplementary Figure S7D), the proposed model showed minor improvement in terms of precision (0.06 increase; *P* = 0.827) and AUROC (0.06 increase; *P* = 0.371) and a significant improvement in recall (0.682 increase; *P* < 0.001).

#### Failure analysis and correlation with overall survival

We observed that the model failed to make a tumor detection, and hence any subsequent molecular status classification, for a small number of cases (1 of 189 and 1 of 259 cases in WUSM and EGD sets, respectively; columns marked with “BG” in Supplementary Figure S7B). For the other misclassifications, no discernible patterns could be identified. However, in the 13.7% (17 of 124) 1p/19q noncodeleted cases in WUSM test set that were misclassified as codeleted, we found a predominance of IDH-mut cases compared to IDH-wt (11/17 IDH-mut, 3/17 IDH-wt, 3/17 IDH status unknown). Additionally, certain cases showed typical features of 1p/19q codeletion like frontal location, heterogeneous texture, and cortical infiltration (Figure 4C – case1). Of the 26.2% (17 of 65) 1p/19q codeleted cases in WUSM test set misclassified as noncodeleted, we found five CNS WHO grade 4 glioblastoma cases that were IDH-wt and had low survival (median OS 5.2 months, range 0.1–13.4 months) (Figure 4C - case2). This genetic-histologic combination is more consistent with 1p/19q noncodeletion.

## Discussion

We developed a DL model for classification of IDH mutation and 1p/19q codeletion status that combines prior clinical knowledge and imaging features through a hybrid CNN architecture. To the best of our knowledge, the proposed method has been validated on the largest dataset till date, obtained from one clinical and seven public sources. The model achieved high accuracy on this heterogeneous dataset and showed excellent generalization on unseen testing data. The code and trained models of this work are available.

Previous studies have explored the association between tumor phenotype and molecular status. Qualitative analyses have examined visual signatures from MRI according to the Visually AcceSAble Rembrandt Images (VASARI) guidelines or the T2-FLAIR mismatch signature.^38^ Quantitative analyses have investigated combining radiomic features and ML.^11,12^ Though ML models have been shown to perform better than visual analysis,^39^ they still require manual intervention due to extensive feature engineering and selection. Hence, they often suffer from lack of reproducibility on new datasets. In contrast to both visual and ML approaches, our CNN-based workflow is completely end-to-end, does not require any manual intervention, automatically learns hierarchical features, integrates readily available clinical information, and shows great generalization on external datasets.

Previous studies have also explored CNN-based approaches for predicting the molecular landscape of gliomas. Our study improves previous work in several ways. First, unlike previous studies^14,16^ with small sample size or lack of external validation, the generalizability of our model was validated on the largest external dataset till date, comprising 968 patient cases from 11 institutions. Second, previous methods often required a previously segmented tumor mask^6,15,18^ or a manually extracted bounding box^16^ around the tumor for classification. In contrast, our model simultaneously detects and segments the glioma and classifies its molecular status. This multi-task approach obviates the requirement of any prior tumor segmentation and enables the model to learn context from multiple related tasks. Third, our model is agnostic to glioma grade and thus moves substantially beyond prior efforts^14,17^ that were limited to specific glioma grades. This facilitates the clinical translation of our model as the tumor grade is unknown in the clinical pre-operative setting. Fourth, objective comparison between different methods is hindered by usage of different datasets and performance metrics. To address this, we performed head-to-head comparisons between our method and two recent approaches.^19,20^

Specifically, we used an independent dataset to explore methodological, computational and performance advantages of the proposed method compared to the works of Voort et al.^20^ and Choi et al.^19^ Our model achieved significantly better overall performance compared to the multi-task CNN method by Voort et al.^20^ As shown by our ablation studies, this is due to our hybrid model’s ability to jointly learn from images as well as knowledge distilled from clinical records and neuro-anatomical information. A hybrid approach was also proposed by Choi et al.^19^ However, their model combined radiomic features with a 2D CNN to predict only IDH status, thus not providing a full classification of the gliomas, and required a separate CNN for tumor segmentation. In comparison with Choi-CNN, the proposed model yielded significantly higher overall performance. This improvement can be attributed to the usage of 2.5D models, which capture a richer spatial context of the brain compared to 2D models, while being computationally efficient. This was also supported by our ablation studies that showed that the 2.5D model performed significantly better than the 2D planar models for both prediction tasks.

In an overall comparison between the IDH and 1p/19q classification performances, we found that the models generally yielded better results for IDH. This is in line with a recent review of radiogenomic studies^11^ that observed a significantly poorer 1p/19q classification performance compared to other molecular subtypes.

For IDH status classification, multiple studies^6,39,40^ have associated IDH-wt gliomas with thick, irregular, and poorly marginated enhancement on T1c scan and IDH-mut gliomas with minimal or no enhancement on T1c, and well-defined tumor margins. There is also evidence^39–41^ of lower age of diagnosis in patients with IDH-mut gliomas compared to IDH-wt. In our study, analysis of cases with misclassified IDH status showed that this existing knowledge of age, tumor phenotype, and OS trends was better aligned with the predicted class than with the ground truth. This alludes to possible errors in the histopathological assessment of the tumor molecular status originating from variability in cutoff values used to determine IDH status in immunohistochemistry (IHC) evaluations,^42^ heterogeneity of staining in IHC leading to partial uptakes,^43^ or heterogeneity in samples where only a fraction of tumor cells have IDH1-R132H expression.^44^

For 1p/19q status classification, several 1p/19q codeleted cases that were misclassified as noncodeleted were in fact glioblastomas with low OS. This suggests possible histopathological false-positive assessment for these cases caused by a partial 1p/19q codeletion^45^ being misclassified by the fluorescence in situ hybridization (FISH) technique due to its inability to distinguish partial from whole-arm deletions. Partial deletions, specifically interstitial and terminal 1p deletions, have been suggested to be particularly common in glioblastomas and are known to confound the FISH assay.^46^

Besides these possible errors, other possible explanations for misclassifications include previous nonclassified glioma subtypes included in the newer WHO 2021 classifications.^2^ However, the current histopathological and molecular assessment relies on invasively and locally obtained tissue samples. In contrast, the proposed work offers several advantages. First, our workflow can ­perform a noninvasive pre-operative determination of ­molecular status that can inform clinical decision-making and lead to a better OS.^47,48^ Second, the proposed model can enable fast, cost-effective tumor characterization that can be particularly useful in low-resource settings. Third, it can be useful for patients with certain risk factors for biopsy (eg, due to old age or other neurological conditions) or tumors which are difficult to operate on (eg, due to location in eloquent brain). Fourth, besides pre-operative treatment planning, this model can be used for repeated evaluation of the molecular status, thus allowing longitudinal characterization of tumor without any associated invasive interventions. Overall, in this emerging era of precision diagnostics, this workflow can drive personalized treatment planning by streamlining molecular characterization of gliomas.

There are certain limitations in this study that merit discussion. First, studies have shown the importance of tumor blood flow information from perfusion imaging,^49^ or detection of 2-HG within tumor through MR-spectroscopy^50^ in IDH prediction. However, in this work, we included only routine MR sequences as advanced sequences are often not included in clinical tumor protocol. This makes clinical translation of our model easier, while allowing us to leverage a much bigger dataset to train and validate our model. Second, we have included IDH and 1p/19q in this study as they are the two most important factors in classification of glioma since the WHO 2016 guidelines.^1^ However, as per the recent WHO 2021 guidelines,^2^ knowledge of telomerase reverse transcriptase (TERT) promoter methylation, epidermal growth factor receptor (EGFR) gene amplification, and combined chromosome 7 gain/chromosome 10 loss status are also required for classifying IDH-wt grade 2/3 gliomas into “Glioblastoma, IDH-wildtype” or “NEC (not elsewhere classified)” classes. These markers could not be included in this study due to the lack of availability, but future work should evaluate the possibility of predicting them based on pre-operative MRI.

In conclusion, we developed a CNN model that can classify IDH mutation and 1p/19q codeletion status from pre-operative structural MR sequences. The model can be extended to predict other molecular alterations that are associated with specific phenotypical signatures on MR images. The network provides an important step towards developing an artificial intelligence–augmented neuro-oncology workflow that can pre-operatively predict tumor behavior and assist treatment planning leading to better outcomes.

## Supplementary Material

vdad023_suppl_Supplementary_Material
