# Supplementary material for: MRI-based classification of IDH mutation and 1p/19q codeletion status of gliomas using a 2.5D hybrid multi-task convolutional neural network
Source: Neurooncol Adv. 2023 Mar 5;5(1):vdad023. doi: 10.1093/noajnl/vdad023 (PMC10162113; doi:10.1093/noajnl/vdad023)
Supplement: vdad023_suppl_Supplementary_Material [file vdad023_suppl_supplementary_material.docx]

**Supplementary data**

**S1. Supplementary methods**

**S1.1 Ground truth information of datasets**

Genetic and histological data were available for all public sources. For the in-house WUSM dataset, IDH status is determined using immunohistochemistry (monoclonal antibody for R132H) routinely as a first pass which is able to pick up the most common mutation (R132H) in 90% cases. If that is negative, then samples are sent for targeted next-generation sequencing (tNGS) to pick up non-canonical variants. For determining 1p/19q status, Fluorescence in situ hybridization (FISH) was performed on paraffin-embedded tissue with locus-specific commercial probes (Abbott Molecular, Des Plaines, IL) localizing to 1p36, 1q25, 19p13 and 19q13.

Expert-annotated tumor segmentation masks were available for the BraTS, LGG-1p/19q, Ivy GAP, and EGD datasets. These multi-class tumor segmentation masks comprised edema, non-enhancing/necrotic tumor core and enhancing tumor, which were combined to define the whole tumor class.

**S1.2 Data acquisition parameters**

The most common parameters along with their percentage in the corresponding datasets are as follows: magnetic field strength: 1.5T in BraTS 2018 (57%), 1.5T in BraTS 2019 (53%), 1.5T in EGD (77%), 3T in Ivy GAP (71%), 1.5T in LGG-1p/19q (95%), 1.5T in TCGA-GBM (64%), 3T in TCGA-LGG (71%), 3T in WUSM (58%); scanner manufacturer: General Electric (GE) in BraTS 2018 (61%), GE in BraTS 2019 (49%), Siemens in EGD (46%), GE in Ivy GAP (91%), GE in LGG-1p/19q (93%), GE in TCGA-GBM (34%), GE in TCGA-LGG (71%), Siemens in WUSM (94%); T1c slice thickness: 2.0–3.0 mm in BraTS 2018 (41%), BraTS 2019 (37%), LGG-1p/19q (82%), TCGA-GBM (31%), and 1.0–2.0 mm in Ivy GAP (68%), TCGA-LGG (68%), ≤1.0 mm in WUSM (58%); T2 slice thickness: 4.0–5.0 mm in BraTS 2018 (62%), BraTS 2019 (73%), TCGA-GBM (53%),1.0–2.0 mm in Ivy GAP (60%), TCGA-LGG (58%), WUSM (69%), and 2.0–3.0 mm in LGG-1p/19q (81%); FLAIR slice thickness: 4.0–5.0 mm in BraTS 2018 (50%), BraTS 2019 (55%), TCGA-GBM (55%), TCGA-LGG (50%), WUSM (93%), and 2.0–3.0 mm in Ivy GAP (54%). No acquisition parameters except scanner manufacturer and magnetic field strength were known for the EGD dataset.

**S1.3** **Data pre-processing and feature extraction**

Scans from BraTS^1–3^ and Ivy GAP^4^ datasets were already registered to the SRI24 anatomical atlas^5^, resampled to 1-mm^3^ isotropic resolution and skull-stripped. For consistency, raw data from TCGA-GBM, TCGA-LGG, LGG-1p/19q, and WUSM were pre-processed using the Integrative Imaging Informatics for Cancer Research: Workflow Automation for Neuro-oncology (I3CR-WANO)^6^ framework. Similar to the BraTS pre-processing protocol, I3CR-WANO involves reorientation of all scans to the left-posterior-superior (LPS) orientation, rigid co-registration of T2 and FLAIR scans to the T1c scan on a per-subject basis, rigid registration of T1c to the SRI24 atlas^5^ to obtain the affine transformation from patient space to atlas space, and finally applying this transformation to all co-registered images. The data from EGD had already been registered to the ICBM 2009a nonlinear MNI152 atlas^7,8^. Thus, we linearly registered this data to the SRI24 space using an affine transformation from the MNI152 to SRI24 atlas computed using I3CR-WANO. This same transformation was applied on the accompanying tumor segmentations using a nearest-neighbor interpolation. Subsequently, for each patient, all the scans were skull-stripped. The only difference between the BraTS pre-processing protocol and I3CR-WANO was in this step as we used the Robust Brain Extraction (ROBEX)^9^ tool for this purpose instead of the Brain Mask Generator (BrainMaGe) tool^10^ used in BraTS. Nevertheless, to identify any potential differences in the classification results originating from the differences in pre-processing protocol, we compared the results from I3CR-WANO and BraTS pre-processing protocol (see Supplementary data S2.7). After skull-stripping, image intensities within the brain were normalized to zero mean and unit variance after excluding intensities below the 5th and above the 95th percentile. Finally, all images were downsampled to 128x128x128 before being fed into the network.

The age information was directly extracted from patient records. The tumor location features were extracted using the FSL ‘Atlasquery’ tool after registering the tumor segmentation mask to the “MNI structural atlas” provided with FSL. The specific steps followed for performing Atlasquery were as follows:

- A transformation from SRI24 to MNI152 space (MNI152_T1_1mm_brain.nii.gz, provided with FSL) is calculated using FSL linear registration (FLIRT)
- The tumor mask in SRI24 atlas space is registered to MNI152 space by applying the above transformation using nearest neighbor interpolation
- The tumor mask in MNI152 space is fed to atlasquery (<https://open.win.ox.ac.uk/pages/fsl/fslpy/_modules/fsl/scripts/atlasq.html>) and the tumor location is queried with respect to the "MNI Structural Atlas" (provided with FSL). This generates as output nine probability values corresponding to the tumor being in one of the following nine brain regions: caudate, cerebellum, frontal lobe, insula, occipital lobe, parietal lobe, putamen, temporal lobe, and thalamus.

FSL atlasquery can take as input either a co-ordinate (single voxel) or an entire mask and performs a structural lookup to return the probability of that single voxel or average probability of an entire mask being a member of the different labelled regions within a reference atlas. So, to query the location of a tumor, we can either interrogate the location of a single point of interest of the tumor (e.g., center of mass) with respect to the reference atlas or we can interrogate the location of the entire mask. In our case, we chose to interrogate the entire mask. Upon query, atlasquery returned a soft probability mask to denote the probability of the entire mask being a member of the different labelled regions within the reference atlas. This allowed us to coarsely capture the information regarding the spread of the tumor i.e., if a tumor is restricted within one anatomical region or if it has spread to multiple regions.

**S1.4 Model architecture and hyperparameters**

The networks used in this study are based on the Mask RCNN architecture^11^, built on top of a Residual Network-101 (ResNet-101)-Feature Pyramid Network (FPN) backbone^12,13^, followed by a region proposal network (RPN), a layer for alignment of the region-of-interests (ROI-Align layer), and two network heads for classification/detection and segmentation (Supplementary Figure S6). To incorporate clinical features into the network, the additional features are normalized and concatenated with the CNN feature output in the classification/detection head using a late-fusion branch (Supplementary Figure S6A).

The hyperparameters of the model were determined using five-fold cross-validation. For this purpose, we split the training data into five folds using a stratified sampling strategy to maintain the original ratio of different image classes in every fold. Next, we trained the model on four folds of data (80% of training data) and validated its performance on the held-out fold (20% of the training data). This process was repeated for each fold in a round-robin fashion. Different sets of hyperparameters were chosen using a random search strategy^14^. The set of hyperparameters that yielded the best cross-validation results was then selected. Next, the model was trained on 100% of the training data using this best set of hyperparameters. The resulting model was subsequently used for prediction on the internal and external test sets.

The network was trained in two stages for a total of 200 epochs. In the first stage, the network head (entire network except backbone, i.e., RPN, classifier and mask heads of the network) was trained for 75 epochs. In the subsequent second stage, the entire network was trained for 125 epochs. We used a batch-size of 4, a learning rate of 0.001, a Stochastic Gradient Descent (SGD) optimizer with a momentum of 0.9 and gradient clipping at 5.0. An L2-regularization of 1x10^-4^ was used in all trainable layers except the batch-normalization layers. To prevent the network from overfitting, the data were augmented during training using mirroring along the vertical axis and random rotations with probabilities of 0.5. To address class-imbalance, we used a class-weighted softmax cross-entropy loss in the classification head of the model.

**S1.5 Model training and testing strategies**

Our training strategy involves feeding the proposed network with slices extracted from each subject’s T1c, T2, and FLAIR scans (only T1c and T2 in case of 1p/19q) along with the prior knowledge features. The subject’s multiclass tumor segmentation mask is used in three distinct ways. First, the whole tumor mask itself is used as ground-truth for segmentation. Second, the bounding box ground-truth for detection is deduced by calculating the smallest rectangle that encloses the mask. Third, the mask is used to inform the extraction of the 2D input slices from the 3D volumes as follows. First, the edema part of the tumor is excluded and the slice with the biggest TC area is selected (let n be the index of this slice). Subsequently, to naturally augment the training data, we utilize the information from neighboring slices, which share considerable information with the selected n-th slice. Specifically, we select two additional sets of slices by extracting from all available modalities the (n+2)-th slice and the (n–2)-th slice, respectively. Finally, these three sets of slices (i.e., (n+2)-th, n-th and (n-2)-th sets) are used as three different samples per subject, thus naturally increasing the training sample size by a factor of 3.

During testing, for each subject, all slices are extracted from available modality scans (T1c, T2, FLAIR for IDH; T1c, T2 for 1p/19q) from the three orthogonal planes. Next, for each 2D network, all slices of the corresponding plane, along with the prior knowledge features, are fed into the network. This way the entire 3D information is captured, which obviates the requirement of any prior tumor segmentation produced through manual or automatic methods. For each set of slices, the network performs three operations – detection of the tumor using a bounding box, segmentation of the whole tumor, and classification of the molecular status of the tumor. Upon running the network over all sets of slices, the classification result of the corresponding plane is determined using majority-voting across all slices where a tumor has been detected.

Once this process is repeated for axial, coronal, and sagittal planes, three classification outcomes are obtained for each input data. To determine the final classification result, these three predictions from the three planar models are ensembled using majority-voting.

The code has been implemented in Python 3.6.2, using the Keras 2.2.5 and Tensorflow-gpu 1.12.0 libraries.

**S1.6 Ablation studies**

To determine the importance of prior knowledge features in improving the model performance, ablation studies were performed to compare performances between four network schemes viz. CNN without any prior knowledge feature (i.e., conventional CNN), CNN with ‘patient age at diagnosis’ feature (‘CNN+age’), CNN with ‘anatomical tumor location’ feature (‘CNN+loc’), and CNN with both of these features (‘CNN+age+loc’). The network scheme with the best performance among these four combinations was selected for the subsequent step.

Once the network scheme was determined, ablation studies were performed within the scheme to compare the proposed 2.5D model, which aggregates information from all three planes to individual models trained on axial, sagittal, coronal planes (planar models). This was done to determine if the aggregated information from three planes improves model performance. Additionally, the 2.5D model was compared to a 3D implementation to assess differences in computational efficiency and classification performance (see Supplementary data S2.5).

This whole process was repeated separately for the IDH and 1p/19q classification tasks.

**S1.7 Baseline methods for comparison**

The performance of the proposed model was compared to two baseline pre-trained models: (i) a multi-task U-net model by Voort et al.^15^ (referred to as “Voort-CNN”) for both IDH and 1p/19q prediction tasks, (ii) a CNN-radiomics hybrid model by Choi et al.^16^ (referred to as “Choi-CNN”) for only the IDH prediction task. To ensure a fair comparison, the models were compared only on the WUSM test set because the EGD dataset had been used as training data for Voort-CNN in the original paper^15^.

The Voort-CNN model requires pre-contrast T1-weighted (T1w) MRI scans along with T1c, T2, and FLAIR sequences for prediction. Based on this requirement, the original WUSM test sets for IDH prediction (n = 337) and 1p/19q prediction (n = 187) were filtered to smaller “WUSM-IDH-4modalities” (n = 261) and “WUSM-1p/19q-4modalities” (n = 129) subsets, respectively. Results for Voort-CNN were obtained using the trained model provided by the authors as the “svdvoort/prognosais_glioma:1.0.2” docker.

The Choi-CNN model had the same modality requirements as the proposed model and could be evaluated on the same WUSM test set (n = 337). This model had been trained for only IDH prediction [26], and hence the performance could not be compared for 1p/19q prediction. Results for Choi-CNN were obtained using the trained model provided by the authors as a github codebase (<https://github.com/yoonchoi-neuro/automated_hybrid_IDH>).

**S1.8 Statistical analysis**

For AUROC and AUPRC, the 95% confidence intervals (CI) were calculated using a 1000-sample bootstrapping method, i.e., by resampling the prediction scores 1000 times with replacement, calculating the scores for each of those 1000 instances and taking the 5th and 95th percentile values from the sample of 1000 values. The statistical analyses and the visualization of the results were performed using scikit-learn, numpy, pandas, seaborn, and lifelines libraries in Python 3.6.2, and the DTComPair and pROC packages in R, version 4.1.0 (<https://www.rproject.org/>). For all statistical analyses, the threshold for statistical significance was set to *P* < .05.

**S1.9 Misclassifications and survival analysis**

For both IDH and 1p/19q classification tasks, we attempted to identify recurring patterns in the cases misclassified by the best-performing models. Prototypical 1p/19q codeleted gliomas have often been associated with frontal predominance^17,18^ and heterogeneous texture^17,19^. For IDH mutation, studies^20–22^ have associated IDH-wt gliomas with irregular enhancements and ill-defined tumor margins, while IDH-mut gliomas with minimal or no enhancement and well-defined tumor margins. We have analyzed the misclassified cases in the light of these previously established phenotypical and prognostic characteristics. We further hypothesized that the misclassified IDH-wt cases that have IDH-mut like phenotype will have better OS. We analyzed these cases by comparing the groups of misclassified cases (i.e., IDH-wt predicted as IDH-mut and vice versa) in terms of OS.

Additionally, the validity of our model in classifying gliomas into WHO 2016 subtypes as well as WHO 2021 subtypes was evaluated separately. For WHO 2016 subtypes, each case in the dataset was classified according to the WHO 2016 guidelines^23^: (i) IDH-mutated, 1p/19q codeleted, grade 2/3 glioma as oligodendroglioma, (ii) IDH-mutated, 1p/19q non-codeleted, grade 2/3 glioma as IDH-mutated astrocytoma, (iii) IDH-wildtype, 1p/19q non-codeleted, grade 2/3 glioma as IDH-wildtype astrocytoma, (iv) IDH-mutated, grade 4 glioma as IDH-mutated glioblastoma, and (v) IDH-wildtype, grade 4 glioma as IDH-wildtype glioblastoma. For WHO 2021 subtypes, each case in the dataset was classified according to the WHO 2021 guidelines^24^: (i) Astrocytoma, IDH-mutant, (ii) Oligodendroglioma, IDH-mutant, and 1p/19q-codeleted, and (iii) Glioblastoma, IDH-wildtype. In all cases, the glioma grade information was obtained from the clinical records. Based on the ground truth molecular status and predicted molecular status of these cases, the cases were placed into different groups and their OS was compared using Cox regression analysis. We hypothesized that the groups based on ground truth molecular status and the corresponding groups based on predicted molecular status will have no significant difference in terms of OS.

## S1.10 Repeatability of results

Considering the randomness involved in DL approaches resulting in non-deterministic results, we have repeated the experiments with the best performing models of both IDH and 1p/19q five times in addition to the reported results and compared the AUROC and AUPRC values across all runs to determine any differences in performance (see Supplementary Results S2.8).

**S2. Supplementary results**

**S2.1 Dataset characteristics**

For the IDH subset, the internal (*P* = 0.041), WUSM (*P* < 0.001) and EGD (*P* = 0.002) test sets differed significantly in terms of age compared to the training data. In terms of sex, the internal test and training data had no significant difference (*P* = 0.483). However, the WUSM (*P* = 0.041) and EGD (*P* = 0.026) test sets differed significantly compared to training set, with a higher prevalence of male subjects. In terms of IDH status, the internal (*P* = 0.283) and EGD (P = 0.176) test sets showed similar distributions compared to the training data. However, the WUSM test set differed significantly with a much lower prevalence of IDH-mut cases compared to the training set (26.11% vs. 40.81%; *P* < 0.001). In terms of tumor grade, the internal (*P* < 0.001), WUSM (*P* < 0.001), and EGD (*P* < 0.001) test sets differed significantly compared to the training set. Compared to the training set, the internal test set had a much higher percentage of grade 4 gliomas (74.19% vs.46.64%), and lower percentage of grade 3 gliomas (8.06% vs.26.46%). Similar trends were observed in the WUSM (grade 3 13.95%, 4 65.88%) and EGD (grade 3 6.82%, 4 60.24%) test sets.

For 1p/19q subset, the age of subjects in the internal test set was significantly higher than those in the training set (56 years vs.48 years; *P* = 0.001). However, the WUSM (*P* = 0.828) and EGD (*P* = 0.861) test sets showed no significant difference. In terms of sex, the internal test set had a similar distribution (*P* = 0.932) compared to the training set. However, the WUSM (*P* = 0.004) and EGD (*P* = 0.011) test sets differed significantly due to a higher prevalence of male subjects. In terms of 1p/19q status, the WUSM (*P* = 0.994) and EGD (*P* = 0.135) test sets showed a similar distribution compared to the training set. However, the internal test set consisted of a significantly lower percentage of 1p/19q codeleted cases compared to the training set (13.68% vs. 34.77%; *P* < 0.001). In terms of tumor grade, the internal (*P* < 0.001), WUSM (*P* < 0.001), and EGD (*P* < 0.001) test sets differed significantly compared to the training set.

**S2.2 Ablation studies** **for IDH mutation status prediction**

**S2.2.1 Ablation studies with and without prior knowledge features**

We evaluated the performance of the conventional CNN, CNN+age, CNN+loc, and CNN+age+loc models in predicting IDH status and found that the CNN+age model performed significantly better than other models. In comparison, the rest of the models had a performance drop, especially in terms of recall on the WUSM and EGD sets (Supplementary Table S3). This was more pronounced in the CNN and CNN+loc models (P < 0.05 for drop in recall on both WUSM and EGD), demonstrating the importance of age information in the prediction of IDH status. The CNN+age+loc model had a significant drop in precision in the WUSM (P = 0.006) and EGD sets (P < 0.001). Overall, the CNN+age model performed the best and was used for the subsequent ablation study.

**S2.2.2 Ablation studies between 2.5D and planar models**

Within the CNN+age model, the ablation study involving the planar models (Supplementary Table S4) showed that capturing volumetric spatial information through a multi-view aggregation step is beneficial. Specifically, we found that the 2.5D model significantly outperformed the axial, coronal, and sagittal models in terms of precision on the WUSM and EGD test sets. There was a minor drop in recall compared to the coronal model. This was found to be insignificant for the internal test set, but significant for WUSM (0.162 decrease; *P* < 0.001) and EGD (0.054 decrease; *P* = 0.011) test sets. However, both the axial and sagittal models were significantly outperformed by the 2.5D model in terms of recall on the EGD set with minor improvements on the internal and WUSM sets as well. Due to this overall better performance, the 2.5D CNN+age model was used for the final IDH mutation classification task.

**S2.3 Misclassification analysis for IDH prediction**

For the WUSM test set, the model misclassified 5.6% (14 of 249) IDH-wt cases as IDH-mut. This included 3 anaplastic astrocytomas, 1 oligodendroglioma, 1 anaplastic oligodendroglioma, and 1 anaplastic oligoastrocytoma – all with relatively high OS (median OS 49.39 months, range 32.4-82.4 months), low age at diagnosis (median 37.5 years, range 26-51.75 years) and phenotype consistent with prototypical IDH-mut gliomas (Figure 4A – case1). We also found a grade-4 glioblastoma located along the dorsal aspect of the midbrain which has radiographic appearance of a low-grade neoplasm with a relatively high OS of 32.4 months (Figure 4A – case2). Of the 20.5% (18 of 88) IDH-mut cases that were misclassified as IDH-wt, we found 3 glioblastoma cases with relatively low OS (median OS 9.17 months, range 4.37-12.7 months), high age at diagnosis (median 74 years, range 72-77 years) and phenotype consistent with prototypical IDH-wt gliomas, with irregular enhancements (Figure 4A – case3). This was also the case for a gliosarcoma (age 74 years), which had a centrally necrotic mass with peripheral enhancement and vaguely defined tumor boundaries and had a low OS of 11.87 months (Figure 4A – case4). Prediction of the gliosarcoma as IDH-wt is also consistent with the 2016 WHO classification system^23^ which includes gliosarcoma under the umbrella of IDH-wt glioblastomas.

For the EGD dataset, of the 11 IDH-mut cases that were misclassified as IDH-wt, three cases comprised scans of coronal acquisition and had very low off-plane resolutions and one case comprised sagittal acquisition with low off-plane resolutions. On a closer inspection, we found that the model made correct predictions for these cases in the plane of acquisition but misclassified them in the other two planes – leading to an overall incorrect prediction. No OS information was available for the EGD dataset and hence survival analysis could not be performed for this data.

**S2.4 Ablation studies for 1p/19q codeletion status prediction**

**S2.4.1 Ablation studies with and without prior knowledge features**

We evaluated the performance of the conventional CNN, CNN+age, CNN+loc, and CNN+age+loc models and found that the CNN+loc model performed the best. Compared to this, the rest of the schemes performed as follows (Supplementary Table S5). The conventional CNN exhibited a significant drop in precision on all three test sets (P < 0.05) and recall on the WUSM test set (P = 0.033). The CNN+age+loc model had a significant drop in precision and recall (P < 0.05) on the EGD set. The CNN+age model showed a minor, statistically insignificant increase in precision for all three datasets (internal: 0.112 increase; P = 0.502; WUSM: 0.084 increase, P = 0.161; EGD: 0.097 increase, P = 0.121) test sets. However, it had a significant drop in recall on the WUSM and EGD test sets. Overall, inclusion of only ‘loc’ information led to a performance improvement. Hence, the CNN+loc model was used for the subsequent ablation study.

**S2.4.2 Ablation studies between 2.5D and planar models**

Within the CNN+loc model, the ablation study involving the planar models (Supplementary Table S6) showed that capturing volumetric spatial information is beneficial. Specifically, we found that the 2.5D model yielded significantly better precision than the axial and sagittal models on all three test sets. Compared to the coronal model, the 2.5D model exhibited a minor drop in precision on the WUSM set (0.058 decrease; *P* = 0.233) but a significant improvement in recall on the EGD set. Overall, the 2.5D model performance was better than the planar models and hence, the 2.5D CNN+loc model was used for the final 1p/19q codeletion classification task.

**S2.5 Ablation studies between 2.5D and 3D model for IDH and 1p/19q**

Our 2.5D implementation is essentially an ensemble of three planar 2D models. In terms of the general requirements of the model, the 3D model is easier to apply compared to a 2D implementation as the 2D implementation requires a prior manual selection of the best slice to be classified. However, due to the slice-by-slice inference performed during testing in our implementation, our proposed 2.5D approach is on par with a 3D implementation in terms of the general requirements of the model.

To compare the computational requirements of the 2.5D and 3D models, the 3D model was implemented using the same hyperparameters as each of the planar 2D models used in the proposed 2.5D approach. All models were tested on an NVIDIA Tesla V100S-32GB GPU. The computational requirements were compared in terms of total number of trainable parameters of the model, training time, and memory footprint. Each of the 2D models in the 2.5D approach had a total of 63,515,812 trainable parameters and took 04 hrs 26 minutes to be trained on GPU, with a maximum memory usage of 14.8 GB. On the other hand, the 3D model had a total of 145,805,104 trainable parameters and could not be run on GPU due to OOM (out of memory) error. The computational burdens of a 3D model have also been reported by Voort et al. in their paper^15^ where the authors had to distribute training over eight GPUs with a batch size limitation of 1 on each GPU, despite using memory management strategies like mixed precision training.

Nevertheless, to test the 3D performance, a lighter version of the 3D model was implemented by reducing the number of trainable parameters. For this purpose, number of filters in the ResNet-101 Feature Pyramid Network (FPN) backbone was reduced. Specifically, in the bottom-up path of the FPN, instead of using 64 as the base number of filters i.e., {64,256,512,1024,2048} number of filters in the different residual blocks across the four stages of ResNet-101, we used 42 as the base number of filters i.e., {42,168,336,672,1344} number of filters in the residual blocks. Additionally, in the top-down pathway of the FPN, instead of using a fixed feature pyramid dimension of 256 as used in the original FPN paper^13^, we used a reduced dimension of 168. Based on this implementation, the number of trainable parameters of the 3D model was reduced to 62,862,320. This lighter 3D model could be trained in 12 hours 21 minutes with a maximum memory usage of 17.04 GB. However, it came at a significant performance drop for both IDH and 1p/19q classification performance (Supplementary Table S7).

## S2.6 Interpretation of quantitative metrics for 1p/19q status classification

This disparity in AUROC and AUPRC for 1p/19q classification in the internal test data can be explained by the severe class-imbalance. The over-represented negative class (i.e., 1p/19q non-codeleted) in this dataset results in a high number of true negative (TN) predictions and in turn, a low false positive rate (FP/FP+TN). This leads to an overly optimistic performance in terms of AUROC. For a similar reason, the accuracy values are also affected and need to be interpreted with caution. On the other hand, the precision-recall curve only addresses the performance on the positive samples (i.e., 1p/19q codeleted) of the dataset and is not affected by the high number of negative samples. This also makes AUPRC a more reliable metric than AUROC under class-imbalance^25^.

## S2.7 Effects of pre-processing protocol on classification performance

To identify any potential differences in the classification results caused by differences in pre-processing protocols, we pre-processed the TCGA and WUSM datasets using the BraTS Pre-processing Pipeline provided by the Cancer Imaging Phenomics Toolkit (CaPTk)^26^. Next, we used the best-performing models for IDH and 1p/19q classification tasks to perform the classification on this data. Subsequently, we compared these results to the results generated using the data pre-processed using the I3CR-WANO protocol using the McNemar test^27^ for precision, the generalized score statistic^28^ for recall, and the DeLong test^29^ for AUROCs. From our analysis, we determined that there were no statistically significant differences in the classification results between data pre-processed using I3CR-WANO and CaPTk (Supplementary Figure S9, Supplementary Table S8).

## S2.8 Analysis of repeatability of results

To check the repeatability of experiments, the experiments corresponding to the best performing models for IDH and 1p/19q were repeated five times in addition to the reported results. For IDH, this was done for the ‘CNN+age’ and for 1p/19q this was done for the ‘CNN+loc’ experiments. From our analysis, we found that the results were indeed non-deterministic i.e., there were minor differences in the values. However, most of the differences were determined to be statistically insignificant (Supplementary Figure S10, Supplementary Table S9). Specifically, for IDH classification, the only statistically significant difference was found between the run2 vs. the reported results on the EGD dataset (AUROC_run2_ = 0.953 vs. AUROC_reported_ = 0.933, P = 0.002). For 1p/19q, no significant differences were found.

**Supplementary figures**

**
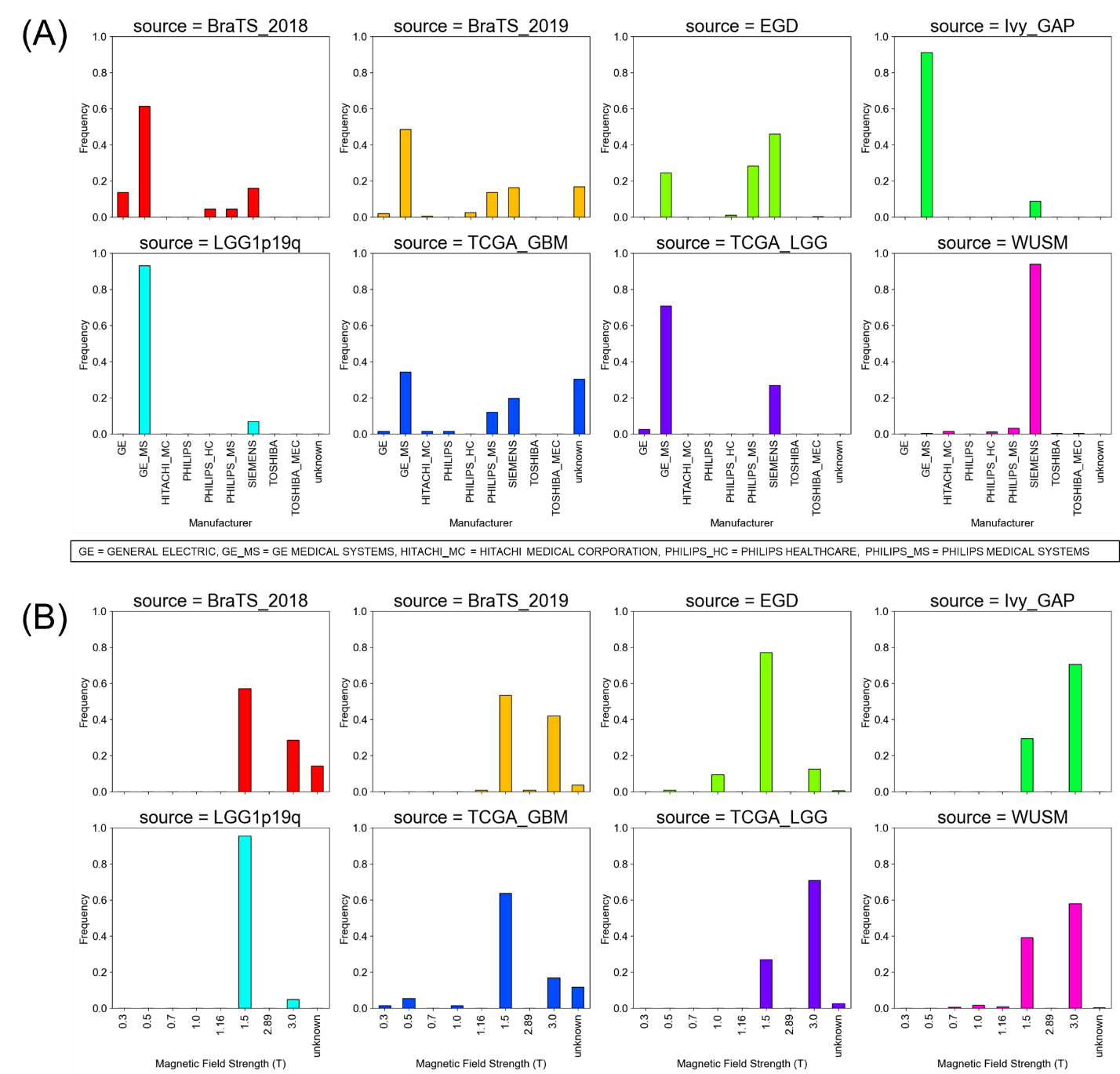
**

**Figure S1.** **(A) Manufacturer, and (B) Magnetic field strength of scans acquired from BraTS 2018, BraTS 2019, EGD, Ivy GAP, LGG 1p/19q, TCGA-GBM, TCGA-LGG, and WUSM datasets. BraTS = Brain Tumor Segmentation challenge, EGD = Erasmus Glioma Database, Ivy GAP = Ivy Glioblastoma Atlas Project, TCGA = The Cancer Genome Atlas, WUSM = Washington University School of Medicine.**


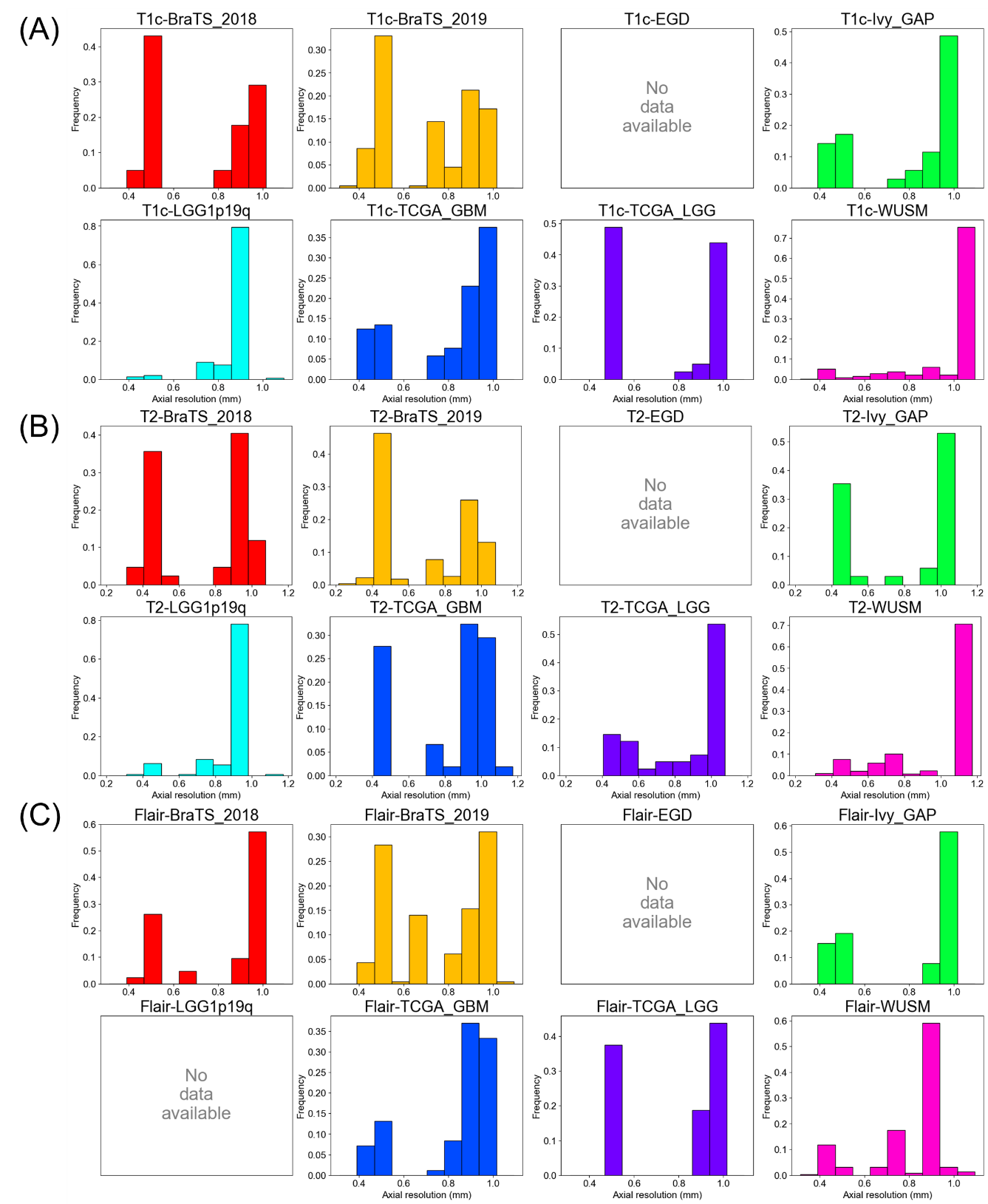


**Figure S2. Axial plane resolution of (A) T1c, (B) T2, and (C) FLAIR scans acquired from BraTS 2018, BraTS 2019, EGD, Ivy GAP, LGG 1p/19q, TCGA-GBM, TCGA-LGG, and WUSM datasets. T1c = post-contrast T1-weighted sequence, T2 = T2-weighted sequence, FLAIR = Fluid Attenuated Inversion Recovery sequence, BraTS = Brain Tumor Segmentation challenge, EGD = Erasmus Glioma Database, Ivy GAP = Ivy Glioblastoma Atlas Project, TCGA = The Cancer Genome Atlas, WUSM = Washington University School of Medicine.**


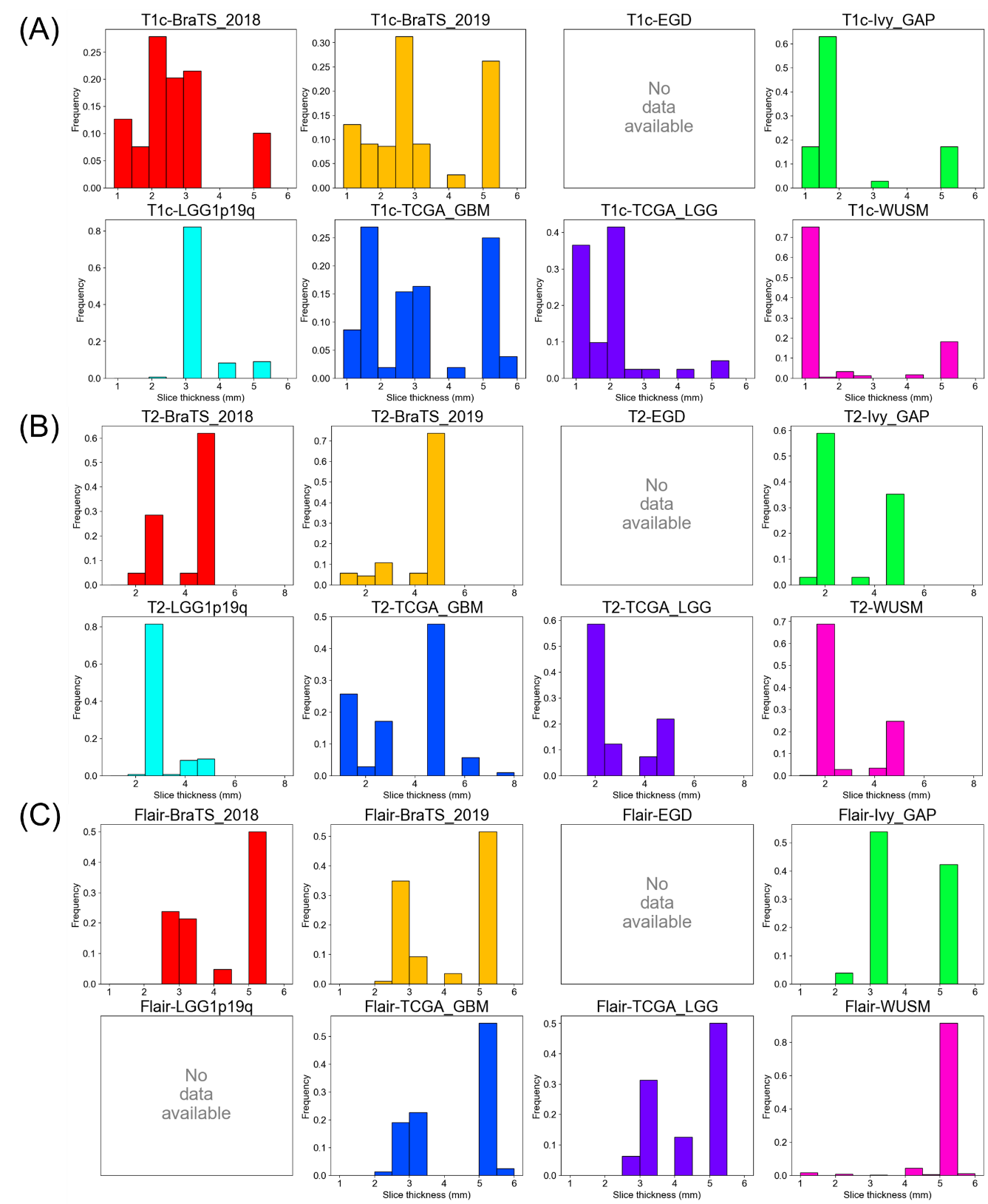


**Figure S3. Slice thickness of (A) T1c, (B) T2, and (C) FLAIR scans acquired from BraTS 2018, BraTS 2019, EGD, Ivy GAP, LGG 1p/19q, TCGA-GBM, TCGA-LGG, and WUSM datasets. T1c = post-contrast T1-weighted sequence, T2 = T2-weighted sequence, FLAIR = Fluid Attenuated Inversion Recovery sequence, BraTS = Brain Tumor Segmentation challenge, EGD = Erasmus Glioma Database, Ivy GAP = Ivy Glioblastoma Atlas Project, TCGA = The Cancer Genome Atlas, WUSM = Washington University School of Medicine.**

**
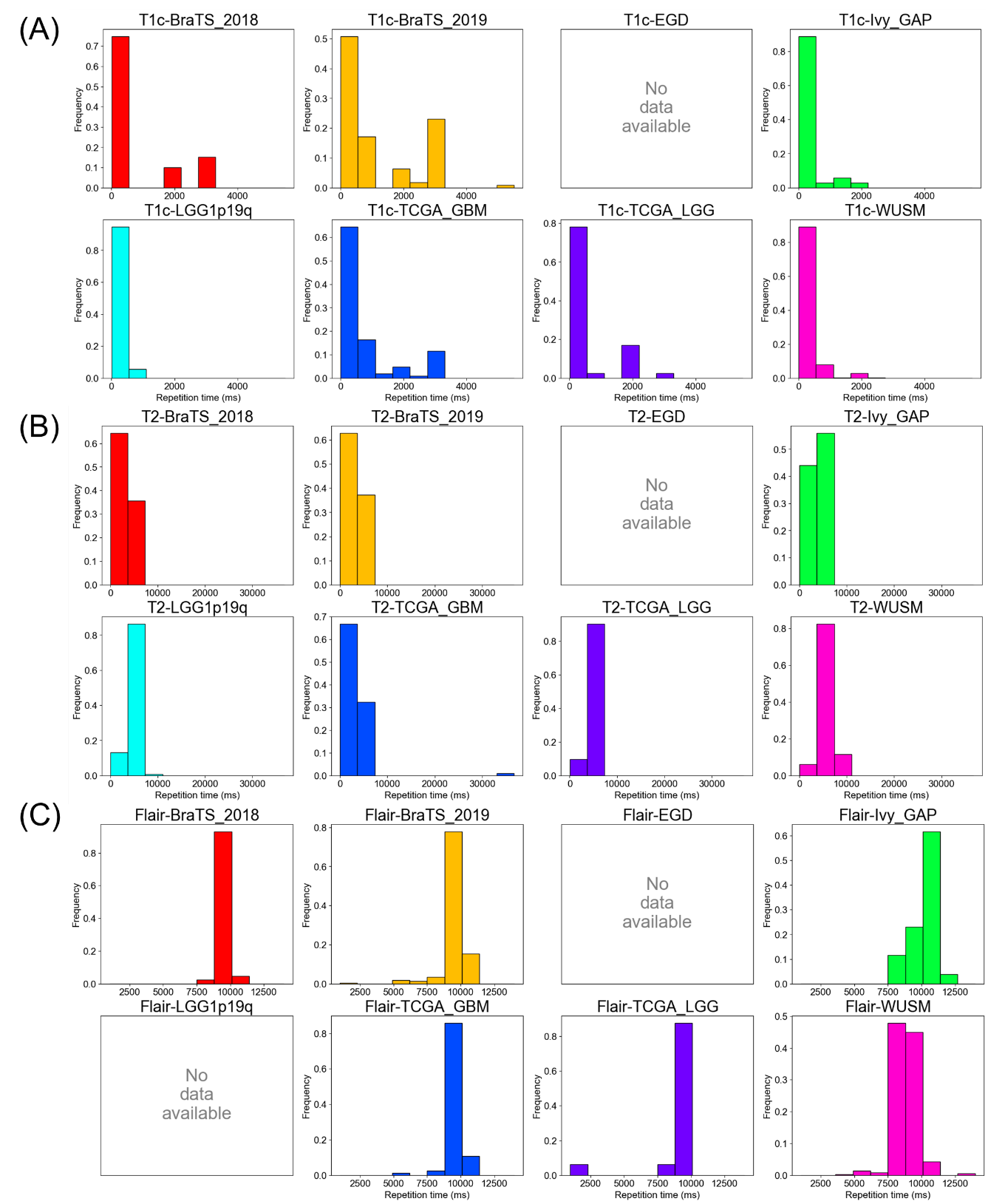
Figure S4. Time of repetition (TR) of (A) T1c, (B) T2, and (C) FLAIR scans acquired from BraTS 2018, BraTS 2019, EGD, Ivy GAP, LGG 1p/19q, TCGA-GBM, TCGA-LGG, and WUSM datasets. T1c = post-contrast T1-weighted sequence, T2 = T2-weighted sequence, FLAIR = Fluid Attenuated Inversion Recovery sequence, BraTS = Brain Tumor Segmentation challenge, EGD = Erasmus Glioma Database, Ivy GAP = Ivy Glioblastoma Atlas Project, TCGA = The Cancer Genome Atlas, WUSM = Washington University School of Medicine.**

**
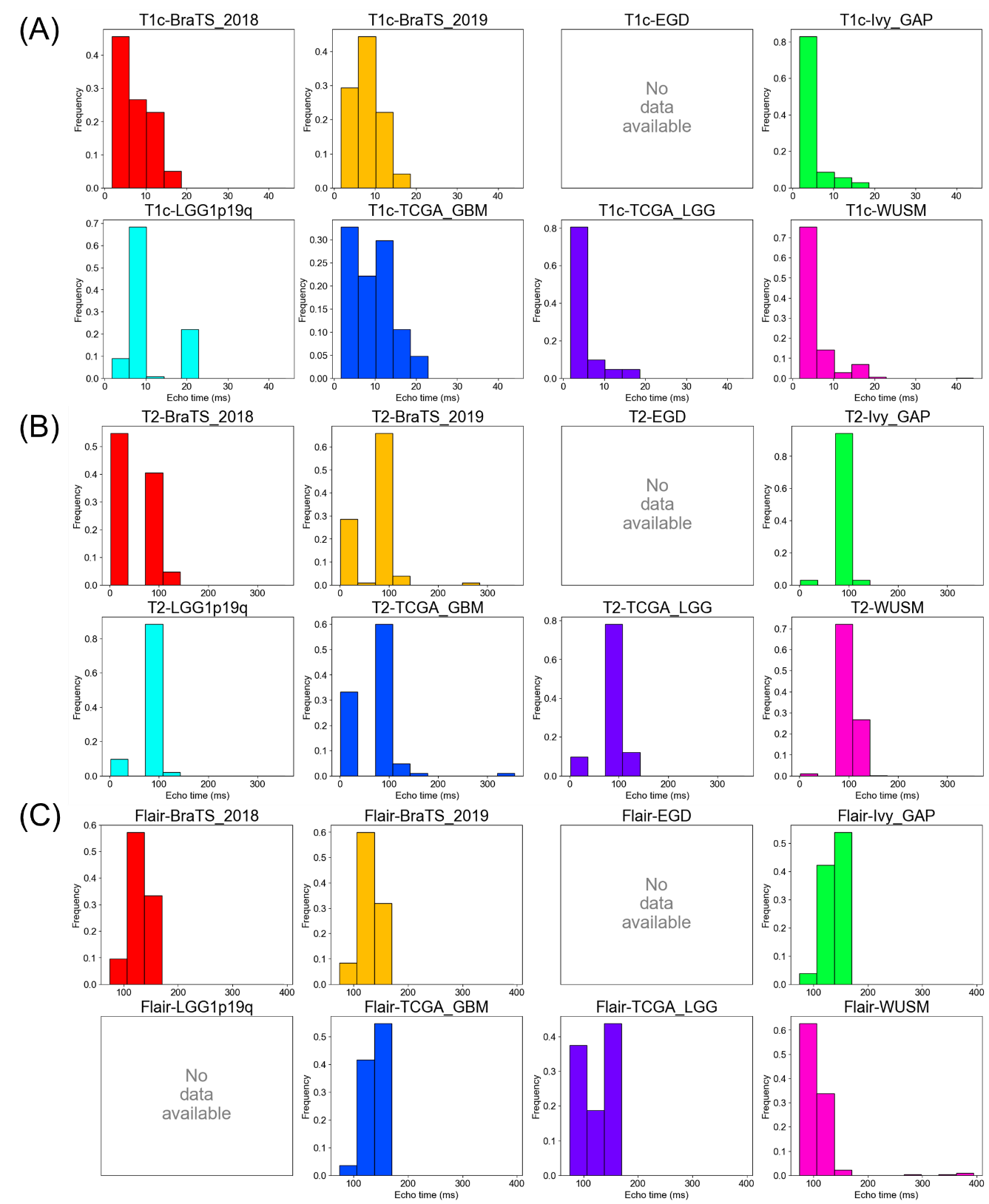
**

**Figure S5. Time of echo (TE) of (A) T1c, (B) T2, and (C) FLAIR scans acquired from BraTS 2018, BraTS 2019, EGD, Ivy GAP, LGG 1p/19q, TCGA-GBM, TCGA-LGG, and WUSM datasets. T1c = post-contrast T1-weighted sequence, T2 = T2-weighted sequence, FLAIR = Fluid Attenuated Inversion Recovery sequence, BraTS = Brain Tumor Segmentation challenge, EGD = Erasmus Glioma Database, Ivy GAP = Ivy Glioblastoma Atlas Project, TCGA = The Cancer Genome Atlas, WUSM = Washington University School of Medicine.**


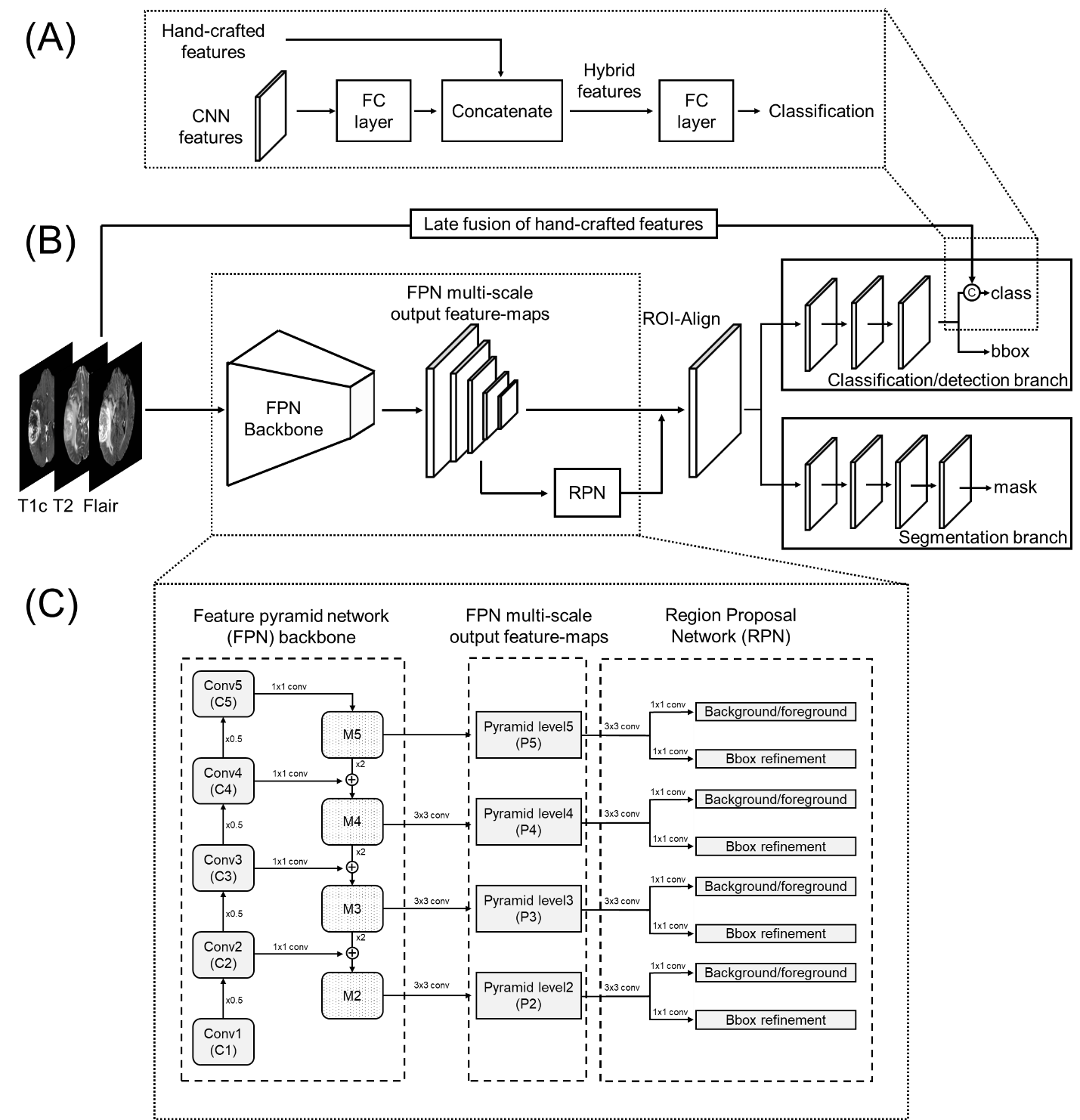


**Figure S6. (A) The late-fusion scheme for integrating prior knowledge features into CNN, (B) the overall hybrid Mask-RCNN architecture, and (C) the detailed architecture of the FPN and RPN. CNN: Convolutional Neural Network, FPN: Feature Pyramid Network, RPN: Region Proposal Network.**


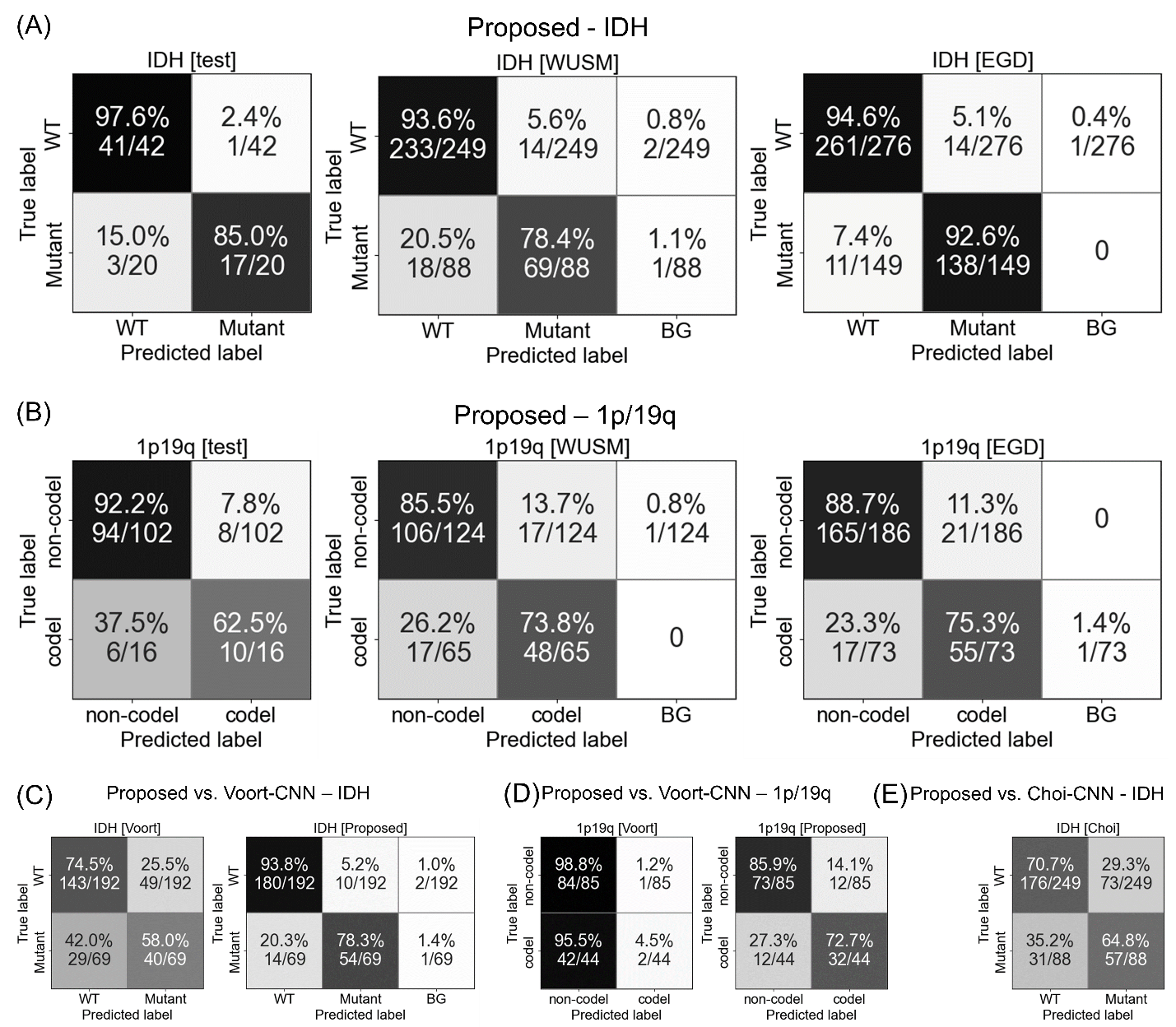


**Figure S7. Confusion matrices for (A) model performance for IDH status prediction, (B) model performance for 1p/19q prediction, (C) comparison of model performance to Voort-CNN**^15^ **for IDH prediction, (D) comparison of model performance to Voort-CNN**^15^ **for 1p/19q prediction, and (E) comparison of model performance to Choi-CNN**^16^ **for IDH prediction.**


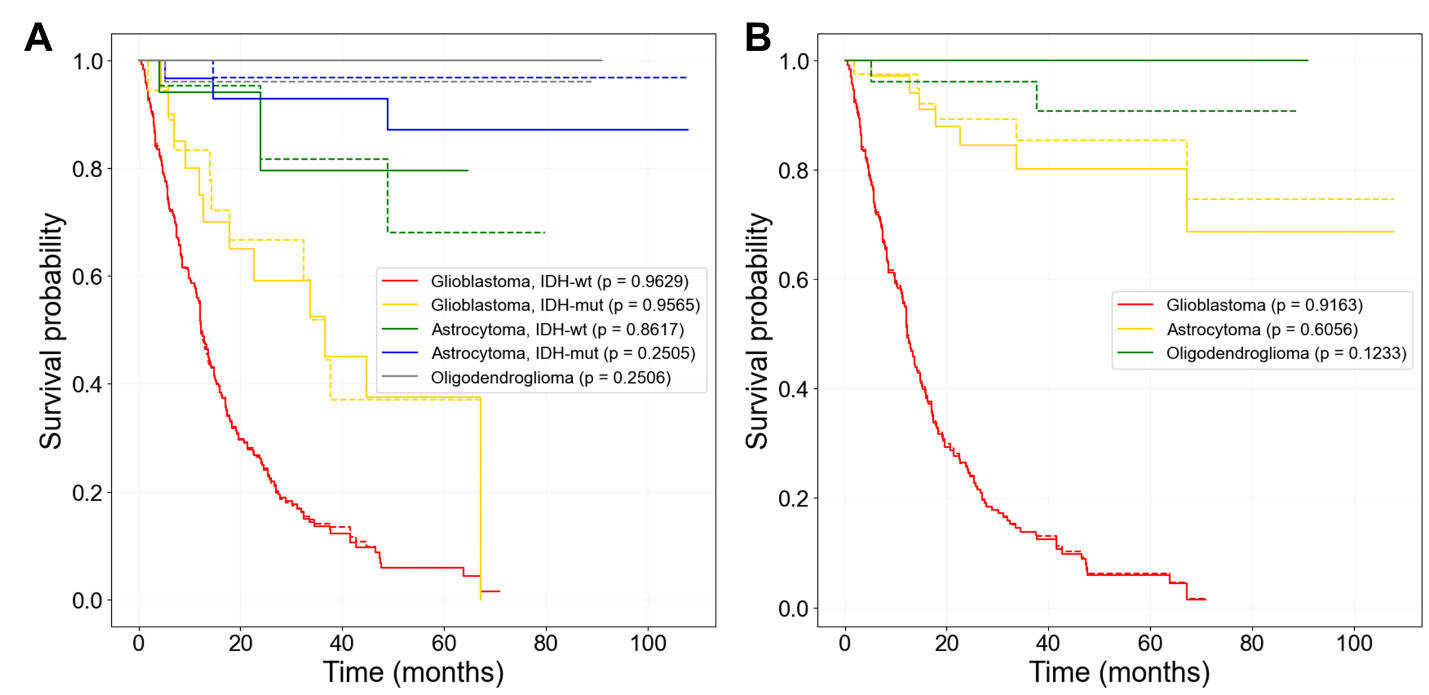


**Figure S8. Kaplan-Meier survival curves characterizing the OS for (A) WHO 2016 glioma subtypes, and (B) WHO 2021 glioma subtypes. For each subtype, the solid and dashed lines characterize the OS based on ground truth and predicted molecular status respectively. The p-values in the parentheses for each subtype (all P > .05) demonstrate that there is no statistically significant difference in the OS values between each ground truth and predicted pair.**


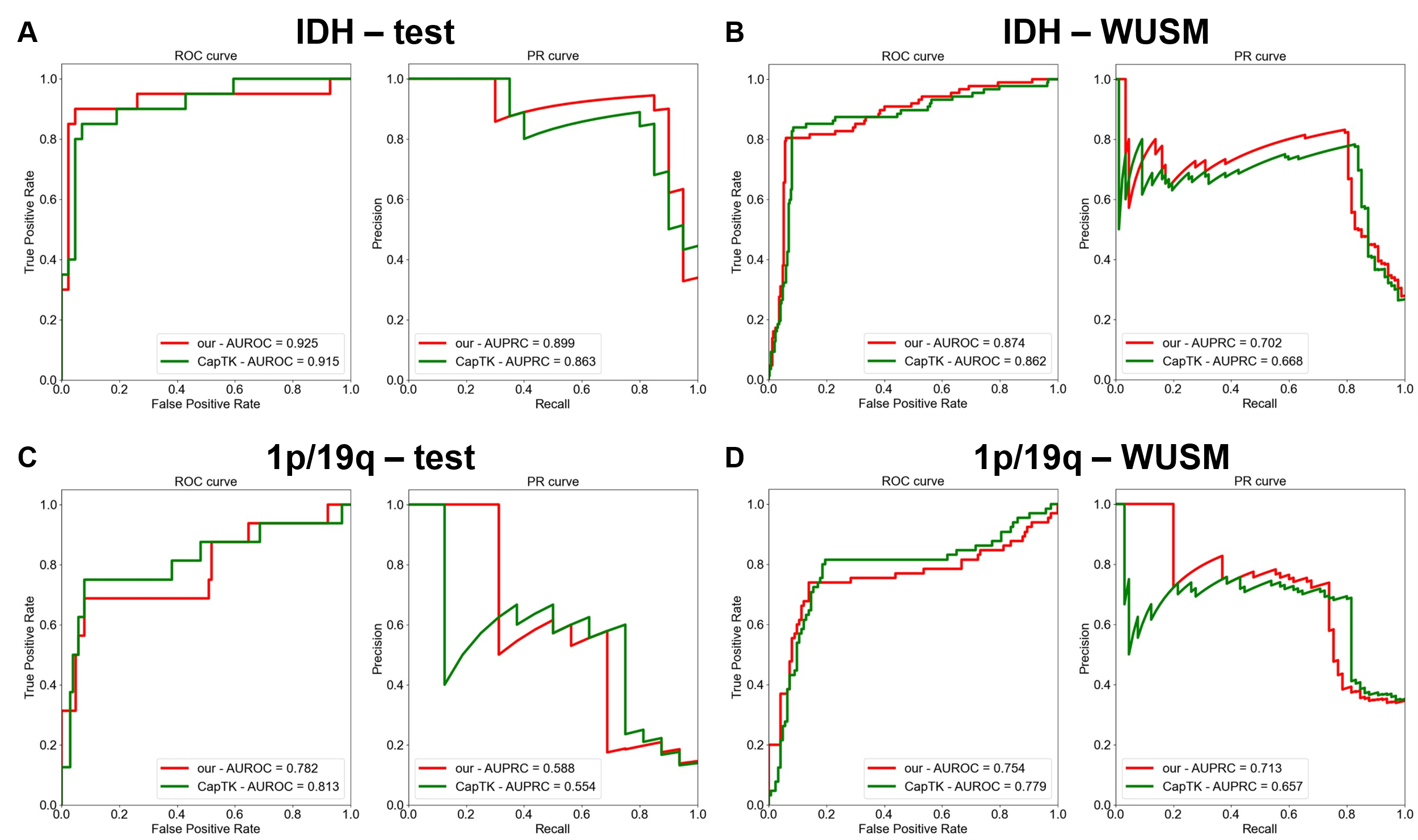
**Figure S9. ROC and PR curves showing the classification performance on data pre-processed by our internal pre-processing protocol (in red) and CaPTk BraTS pre-processing pipeline (in green) for (A) IDH classification on test data, (B) IDH classification on WUSM data, (C) 1p/19q classification on test data, and (D) 1p/19q classification on WUSM data. In all cases, no statistically significant differences were found in the classification performance between the two pre-processing protocols.**


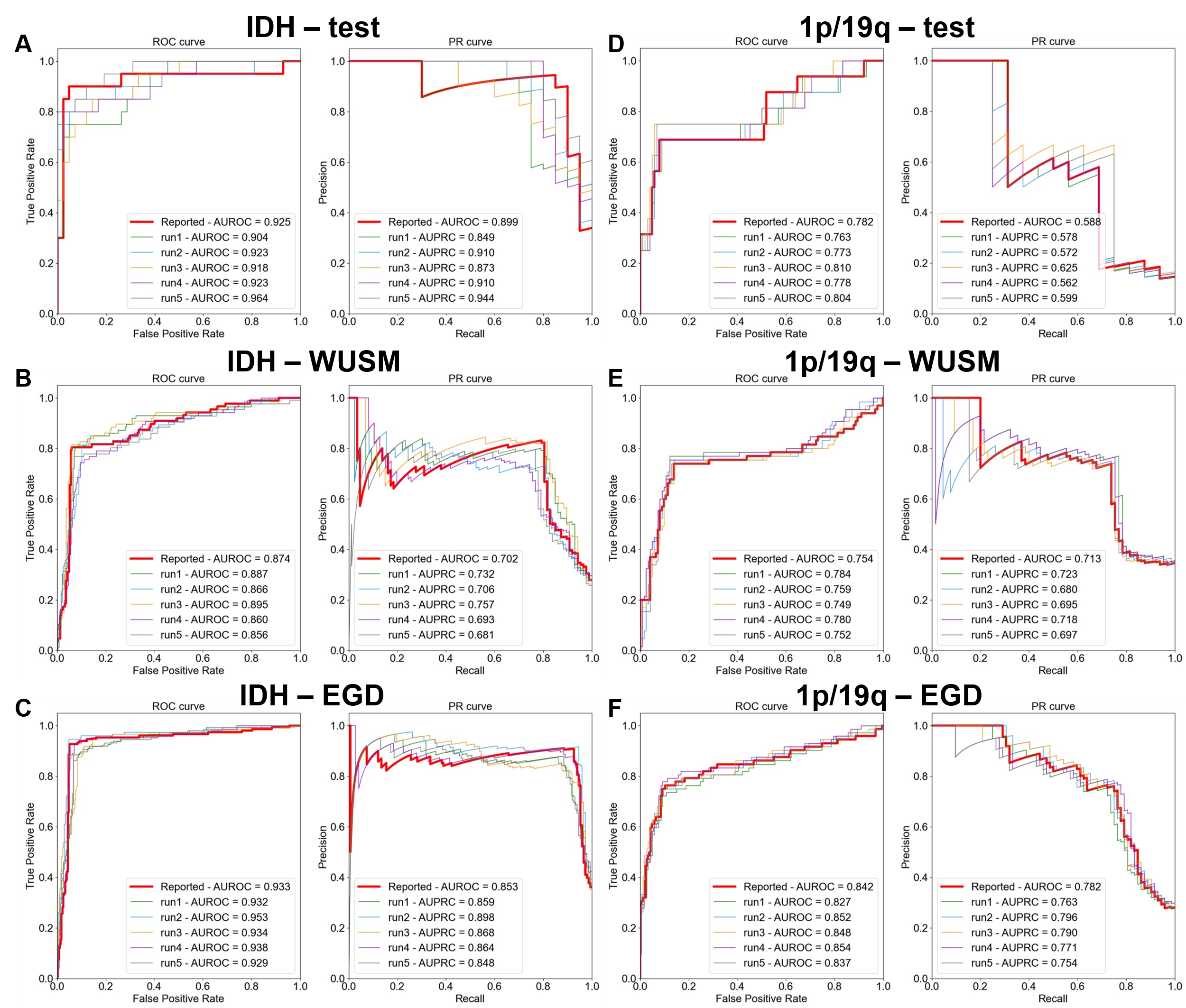


**Figure S10. ROC and PR curves showing the** **repeatability of results for the best performing models for IDH and 1p/19q classification tasks, i.e., the reported results and five additional runs (run1 – run5) for the (A)–(C)** **‘CNN+age’ experiment for IDH classification and (D)–(F) ‘CNN+loc’ experiment for 1p/19q classification, on the test, WUSM, and EGD datasets. No statistically significant differences were found in the classification performance between the different runs except run2 vs. reported for IDH classification on the EGD dataset (AUROC_run2_ = 0.953 vs. AUROC_reported_ = 0.933, P = 0.002).**

**Supplementary tables**

**Table S1. Statistical comparison of performance between proposed model and Voort-CNN and Choi-CNN for prediction of IDH mutation status**

|  | Precision | Difference | P-value | Recall | Difference | P-value | AUROC | Difference | P-value |
| --- | --- | --- | --- | --- | --- | --- | --- | --- | --- |
| Voort-CNN | 0.453 | -0.391 | <0.001 | 0.574 | -0.22 | 0.004 | 0.755 | -0.113 | 0.002 |
| Choi-CNN | 0.438 | -0.393 | <0.001 | 0.644 | -0.149 | 0.028 | 0.704 | -0.17 | <0.001 |

**Table S2. Statistical comparison of performance between proposed model and Voort-CNN for prediction of 1p/19q codeletion status**

|  | Precision | Difference | P-value | Recall | Difference | P-value | AUROC | Difference | P-value |
| --- | --- | --- | --- | --- | --- | --- | --- | --- | --- |
| Voort-CNN | 0.667 | -0.06 | 0.827 | 0.045 | -0.682 | <0.001 | 0.673 | -0.06 | 0.371 |

**Table S3. Statistical comparison of performance between CNN, CNN+loc, CNN+age+loc models with proposed model (CNN+age) for prediction of IDH mutation status**

|  | Precision | Difference | P-value | Recall | Difference | P-value | AUROC | Difference | P-value |
| --- | --- | --- | --- | --- | --- | --- | --- | --- | --- |
| Test |  |  |  |  |  |  |  |  |  |
| CNN | 1 | 0.056 | 0.303 | 0.75 | -0.1 | 0.157 | 0.926 | 0.001 | 0.978 |
| CNN+loc | 0.579 | -0.365 | <0.001 | 0.55 | -0.3 | 0.014 | 0.738 | -0.187 | 0.004 |
| CNN+age+loc | 0.889 | -0.055 | 0.530 | 0.8 | -0.05 | 0.564 | 0.912 | -0.013 | 0.657 |
| WUSM |  |  |  |  |  |  |  |  |  |
| CNN | 0.873 | 0.046 | 0.254 | 0.632 | -0.156 | <0.001 | 0.854 | -0.019 | 0.280 |
| CNN+loc | 0.456 | -0.371 | <0.001 | 0.612 | -0.176 | <0.001 | 0.73 | -0.143 | <0.001 |
| CNN+age+loc | 0.733 | -0.094 | 0.006 | 0.741 | -0.047 | 0.248 | 0.849 | -0.024 | 0.187 |
| EGD |  |  |  |  |  |  |  |  |  |
| CNN | 0.914 | 0.007 | 0.770 | 0.858 | -0.068 | 0.025 | 0.939 | 0.006 | 0.661 |
| CNN+loc | 0.623 | -0.284 | <0.001 | 0.736 | -0.19 | <0.001 | 0.824 | -0.109 | <0.001 |
| CNN+age+loc | 0.79 | -0.117 | <0.001 | 0.865 | -0.061 | 0.039 | 0.909 | -0.024 | 0.049 |

Abbreviations: WUSM, Washington University School of Medicine; EGD, Erasmus Glioma Database; AUROC, area under the receiver operating characteristic curve; CNN, conventional CNN; CNN+loc, CNN with tumor location information; CNN+age CNN with patient age information; CNN+age+loc, CNN with patient age and tumor location information.

**Table S4. Statistical comparison of performance between axial, coronal, sagittal models with proposed model for prediction of IDH mutation status**

|  | Precision | Difference | P-value | Recall | Difference | P-value | AUROC | Difference | P-value |
| --- | --- | --- | --- | --- | --- | --- | --- | --- | --- |
| Test |  |  |  |  |  |  |  |  |  |
| axial | 1 | 0.056 | 0.304 | 0.5 | -0.35 | 0.008 | 0.896 | -0.029 | 0.419 |
| coronal | 0.514 | -0.43 | <0.001 | 0.95 | 0.1 | 0.157 | 0.799 | -0.126 | 0.007 |
| sagittal | 0.762 | -0.182 | 0.074 | 0.8 | -0.05 | 0.317 | 0.88 | -0.045 | 0.276 |
| WUSM |  |  |  |  |  |  |  |  |  |
| axial | 0.732 | -0.097 | 0.023 | 0.605 | -0.186 | <0.001 | 0.841 | -0.033 | 0.119 |
| coronal | 0.347 | -0.482 | <0.001 | 0.953 | 0.162 | <0.001 | 0.808 | -0.066 | 0.004 |
| sagittal | 0.508 | -0.321 | <0.001 | 0.756 | -0.035 | 0.083 | 0.787 | -0.087 | <0.001 |
| EGD |  |  |  |  |  |  |  |  |  |
| axial | 0.815 | -0.092 | <0.001 | 0.682 | -0.244 | <0.001 | 0.888 | -0.045 | <0.001 |
| coronal | 0.505 | -0.402 | <0.001 | 0.98 | 0.054 | 0.011 | 0.849 | -0.084 | <0.001 |
| sagittal | 0.743 | -0.164 | <0.001 | 0.899 | -0.027 | 0.046 | 0.89 | -0.043 | 0.003 |

Abbreviations: WUSM, Washington University School of Medicine; EGD, Erasmus Glioma Database; AUROC, area under the receiver operating characteristic curve.

**Table S5. Statistical comparison of performance between CNN, CNN+age, CNN+age+loc models with proposed model (CNN+loc) for prediction of 1p/19q codeletion status**

|  | Precision | Difference | P-value | Recall | Difference | P-value | AUROC | Difference | P-value |
| --- | --- | --- | --- | --- | --- | --- | --- | --- | --- |
| Test |  |  |  |  |  |  |  |  |  |
| CNN | 0.333 | -0.255 | 0.050 | 0.5 | -0.125 | 0.317 | 0.776 | 0.046 | 0.450 |
| CNN+age | 0.7 | 0.112 | 0.502 | 0.438 | -0.187 | 0.257 | 0.451 | -0.279 | 0.064 |
| CNN+age+loc | 0.3 | -0.288 | 0.025 | 0.375 | -0.25 | 0.102 | 0.55 | -0.18 | 0.100 |
| WUSM |  |  |  |  |  |  |  |  |  |
| CNN | 0.455 | -0.283 | <0.001 | 0.615 | -0.123 | 0.033 | 0.674 | -0.079 | 0.060 |
| CNN+age | 0.822 | 0.084 | 0.161 | 0.569 | -0.169 | 0.022 | 0.654 | -0.099 | 0.057 |
| CNN+age+loc | 0.667 | -0.071 | 0.178 | 0.677 | -0.061 | 0.206 | 0.681 | -0.072 | 0.075 |
| EGD |  |  |  |  |  |  |  |  |  |
| CNN | 0.481 | -0.255 | <0.001 | 0.694 | -0.063 | 0.225 | 0.795 | -0.046 | 0.152 |
| CNN+age | 0.833 | 0.097 | 0.121 | 0.571 | -0.186 | 0.005 | 0.637 | -0.204 | <0.001 |
| CNN+age+loc | 0.581 | -0.155 | 0.010 | 0.614 | -0.143 | 0.018 | 0.648 | -0.193 | <0.001 |

Abbreviations: WUSM, Washington University School of Medicine; EGD, Erasmus Glioma Database; AUROC, area under the receiver operating characteristic curve; CNN, conventional CNN; CNN+loc, CNN with tumor location information; CNN+age CNN with patient age information; CNN+age+loc, CNN with patient age and tumor location information.

**Table S6.** **Statistical comparison of performance between** **axial, coronal, sagittal models with proposed model for prediction of 1p/19q codeletion status**

|  | Precision | Difference | P-value | Recall | Difference | P-value | AUROC | Difference | P-value |
| --- | --- | --- | --- | --- | --- | --- | --- | --- | --- |
| Test |  |  |  |  |  |  |  |  |  |
| axial | 0.333 | -0.223 | 0.016 | 0.562 | -0.063 | 0.564 | 0.602 | -0.12 | 0.131 |
| coronal | 0.444 | -0.112 | 0.285 | 0.5 | -0.125 | 0.317 | 0.67 | -0.052 | 0.500 |
| sagittal | 0.364 | -0.192 | 0.021 | 0.75 | 0.125 | 0.317 | 0.803 | 0.081 | 0.438 |
| WUSM |  |  |  |  |  |  |  |  |  |
| axial | 0.616 | -0.122 | 0.006 | 0.692 | -0.046 | 0.366 | 0.71 | -0.044 | 0.308 |
| coronal | 0.796 | 0.058 | 0.233 | 0.662 | -0.076 | 0.059 | 0.73 | -0.024 | 0.494 |
| sagittal | 0.587 | -0.151 | <0.001 | 0.831 | 0.093 | 0.058 | 0.755 | 0.001 | 0.984 |
| EGD |  |  |  |  |  |  |  |  |  |
| axial | 0.56 | -0.164 | <0.001 | 0.708 | -0.056 | 0.206 | 0.75 | -0.092 | 0.017 |
| coronal | 0.638 | -0.086 | 0.072 | 0.611 | -0.153 | 0.005 | 0.79 | -0.052 | 0.126 |
| sagittal | 0.536 | -0.188 | <0.001 | 0.819 | 0.055 | 0.248 | 0.831 | -0.011 | 0.692 |

Abbreviations: WUSM, Washington University School of Medicine; EGD, Erasmus Glioma Database; AUROC, area under the receiver operating characteristic curve.

**Table S7. Statistical comparison of performance between 3D model with respect to the proposed 2.5D model for** **prediction of IDH mutation and 1p/19q codeletion status.**

|  | Precision | Difference | P-value | Recall | Difference | P-value | AUROC | Difference | P-value |
| --- | --- | --- | --- | --- | --- | --- | --- | --- | --- |
| IDH |  |  |  |  |  |  |  |  |  |
| Test | 0.667 | -0.277 | 0.023 | 0.6 | -0.25 | 0.059 | 0.777 | -0.148 | 0.039 |
| WUSM | 0.637 | -0.194 | <0.001 | 0.667 | -0.126 | 0.022 | 0.825 | -0.049 | 0.109 |
| EGD | 0.75 | -0.158 | <0.001 | 0.805 | -0.121 | 0.002 | 0.835 | -0.098 | <0.001 |
| 1p/19q |  |  |  |  |  |  |  |  |  |
| Test | 0.28 | -0.276 | 0.017 | 0.438 | -0.187 | 0.179 | 0.645 | -0.078 | 0.481 |
| WUSM | 0.486 | -0.252 | <0.001 | 0.523 | -0.215 | 0.002 | 0.614 | -0.14 | 0.004 |
| EGD | 0.522 | -0.204 | <0.001 | 0.686 | -0.071 | 0.297 | 0.73 | -0.108 | 0.015 |

Abbreviations: WUSM, Washington University School of Medicine; EGD, Erasmus Glioma Database; AUROC, area under the receiver operating characteristic curve.

**Table S8.** **Statistical comparison of performance between data pre-processed using the CaPTk BraTS pre-processing protocol and the (in-house) I3CR-WANO pre-processing protocol for prediction of IDH mutation and 1p/19q codeletion status.**

|  | Precision | Difference | P-value | Recall | Difference | P-value | AUROC | Difference | P-value |
| --- | --- | --- | --- | --- | --- | --- | --- | --- | --- |
| IDH |  |  |  |  |  |  |  |  |  |
| Test | 0.842 | -0.102 | 0.285 | 0.8 | -0.05 | 0.564 | 0.915 | -0.01 | 0.800 |
| WUSM | 0.783 | -0.048 | 0.100 | 0.828 | 0.035 | 0.405 | 0.862 | -0.012 | 0.609 |
| 1p/19q |  |  |  |  |  |  |  |  |  |
| Test | 0.6 | 0.021 | 0.660 | 0.75 | 0.062 | 0.317 | 0.813 | 0.031 | 0.399 |
| WUSM | 0.679 | -0.059 | 0.212 | 0.815 | 0.077 | 0.251 | 0.779 | 0.025 | 0.667 |

Abbreviations: WUSM, Washington University School of Medicine; AUROC, area under the receiver operating characteristic curve.

**Table S9. Statistical comparison of performance between five additional runs (run1 – run5) and the reported results for the best performing models for IDH (‘CNN+age’ experiment) and 1p/19q (‘CNN+loc’ experiment) classification tasks to demonstrate repeatability of results.**

|  | IDH | | | 1p/19q | | |
| --- | --- | --- | --- | --- | --- | --- |
|  | AUROC | Difference | P-value | AUROC | Difference | P-value |
| Test |  |  |  |  |  |  |
| run1 | 0.904 | -0.021 | 0.663 | 0.763 | -0.019 | 0.235 |
| run2 | 0.923 | -0.002 | 0.926 | 0.773 | -0.009 | 0.541 |
| run3 | 0.918 | -0.007 | 0.862 | 0.81 | 0.028 | 0.412 |
| run4 | 0.923 | -0.002 | 0.952 | 0.778 | -0.004 | 0.754 |
| run5 | 0.964 | 0.039 | 0.298 | 0.804 | 0.022 | 0.500 |
| WUSM |  |  |  |  |  |  |
| run1 | 0.887 | 0.013 | 0.319 | 0.784 | 0.03 | 0.097 |
| run2 | 0.866 | -0.006 | 0.728 | 0.759 | 0.005 | 0.583 |
| run3 | 0.894 | 0.022 | 0.115 | 0.749 | -0.005 | 0.577 |
| run4 | 0.858 | -0.014 | 0.426 | 0.78 | 0.026 | 0.175 |
| run5 | 0.854 | -0.018 | 0.378 | 0.752 | -0.002 | 0.277 |
| EGD |  |  |  |  |  |  |
| run1 | 0.932 | -0.001 | 0.894 | 0.827 | -0.015 | 0.487 |
| run2 | 0.953 | 0.02 | 0.002 | 0.852 | 0.01 | 0.681 |
| run3 | 0.935 | 0.002 | 0.842 | 0.848 | 0.006 | 0.528 |
| run4 | 0.938 | 0.005 | 0.657 | 0.854 | 0.012 | 0.521 |
| run5 | 0.93 | -0.003 | 0.764 | 0.837 | -0.005 | 0.670 |

**Supplementary references**

1. Menze BH, Jakab A, Bauer S, et al. The Multimodal Brain Tumor Image Segmentation Benchmark (BRATS). *IEEE Trans Med Imaging*. 2015;34(10):1993-2024. doi:10.1109/TMI.2014.2377694

2. Bakas S, Akbari H, Sotiras A, et al. Advancing The Cancer Genome Atlas glioma MRI collections with expert segmentation labels and radiomic features. *Sci Data*. 2017;4(July):1-13. doi:10.1038/sdata.2017.117

3. Bakas S, Reyes M, Jakab A, et al. Identifying the Best Machine Learning Algorithms for Brain Tumor Segmentation, Progression Assessment, and Overall Survival Prediction in the BRATS Challenge. *arXiv Prepr arXiv181102629*. 2018;124. Accessed June 7, 2020. http://arxiv.org/abs/1811.02629

4. Puchalski RB, Shah N, Miller J, et al. An anatomic transcriptional atlas of human glioblastoma. *Science (80- )*. 2018;360(6389):660-663.

5. Rohlfing T, Zahr NM, Sullivan E V., Pfefferbaum A. The SRI24 multichannel atlas of normal adult human brain structure. *Hum Brain Mapp*. 2010;31(5):798-819. doi:10.1002/hbm.20906

6. Chakrabarty S, Abidi SA, Mousa M, et al. Integrative Imaging Informatics for Cancer Research: Workflow Automation for Neuro-oncology (I3CR-WANO). *arXiv Prepr arXiv221003151*. Published online October 6, 2022. http://arxiv.org/abs/2210.03151

7. Fonov V, Evans A, McKinstry R, Almli C, Collins D. Unbiased nonlinear average age-appropriate brain templates from birth to adulthood. *Neuroimage*. 2009;47(47):S102. doi:10.1016/s1053-8119(09)70884-5

8. Fonov V, Evans AC, Botteron K, Almli CR, McKinstry RC, Collins DL. Unbiased average age-appropriate atlases for pediatric studies. *Neuroimage*. 2011;54(1):313-327. doi:10.1016/j.neuroimage.2010.07.033

9. Iglesias JE, Liu CY, Thompson PM, Tu Z. Robust brain extraction across datasets and comparison with publicly available methods. *IEEE Trans Med Imaging*. 2011;30(9):1617-1634. doi:10.1109/TMI.2011.2138152

10. Thakur S, Doshi J, Pati S, et al. Brain extraction on MRI scans in presence of diffuse glioma: Multi-institutional performance evaluation of deep learning methods and robust modality-agnostic training. *Neuroimage*. 2020;220:117081.

11. He K, Gkioxari G, Dollár P, Girshick R. Mask R-CNN. In: *IEEE Transactions on Pattern Analysis and Machine Intelligence*. Vol 42. ; 2020:386-397. doi:10.1109/TPAMI.2018.2844175

12. He K, Zhang X, Ren S, Sun J. Delving deep into rectifiers: Surpassing human-level performance on imagenet classification. In: *Proceedings of the IEEE International Conference on Computer Vision*. Vol 2015 Inter. ; 2015:1026-1034. doi:10.1109/ICCV.2015.123

13. Lin T-Y, Dollár P, Girshick R, He K, Hariharan B, Belongie S. Feature pyramid networks for object detection. In: *Proceedings of the IEEE Conference on Computer Vision and Pattern Recognition*. ; 2017:2117-2125.

14. Bergstra J, Bengio Y. Random search for hyper-parameter optimization. *J Mach Learn Res*. 2012;13(2):281-305.

15. van der Voort SR, Incekara F, Wijnenga MMJ, et al. Combined molecular subtyping, grading, and segmentation of glioma using multi-task deep learning. *Neuro Oncol*. Published online 2022. doi:10.1093/neuonc/noac166

16. Choi YS, Bae S, Chang JH, et al. Fully automated hybrid approach to predict the IDH mutation status of gliomas via deep learning and radiomics. *Neuro Oncol*. 2021;23(2):304-313. doi:10.1093/neuonc/noaa177

17. van der Voort SR, Incekara F, Wijnenga MMJ, et al. Predicting the 1p/19q codeletion status of presumed low-grade glioma with an externally validated machine learning algorithm. *Clin Cancer Res*. 2019;25(24):7455-7462. doi:10.1158/1078-0432.CCR-19-1127

18. Shboul ZA, Chen J, M. Iftekharuddin K. Prediction of Molecular Mutations in Diffuse Low-Grade Gliomas using MR Imaging Features. *Sci Rep*. 2020;10(1):1-13. doi:10.1038/s41598-020-60550-0

19. Batchala PP, Muttikkal TJE, Donahue JH, et al. Neuroimaging-based classification algorithm for predicting 1p/19q-codeletion status in IDH-mutant lower grade gliomas. *Am J Neuroradiol*. 2019;40(3):426-432. doi:10.3174/ajnr.A5957

20. Chang P, Grinband J, Weinberg BD, et al. Deep-learning convolutional neural networks accurately classify genetic mutations in gliomas. *Am J Neuroradiol*. 2018;39(7):1201-1207. doi:10.3174/ajnr.A5667

21. Zhou H, Vallières M, Bai HX, et al. MRI features predict survival and molecular markers in diffuse lower-grade gliomas. *Neuro Oncol*. 2017;19(6):862-870. doi:10.1093/neuonc/now256

22. Eckel-Passow JE, Lachance DH, Molinaro AM, et al. Glioma Groups Based on 1p/19q, IDH , and TERT Promoter Mutations in Tumors . *N Engl J Med*. 2015;372(26):2499-2508. doi:10.1056/nejmoa1407279

23. Louis DN, Perry A, Reifenberger G, et al. The 2016 World Health Organization Classification of Tumors of the Central Nervous System: a summary. *Acta Neuropathol*. 2016;131(6):803-820. doi:10.1007/s00401-016-1545-1

24. Louis DN, Perry A, Wesseling P, et al. The 2021 WHO classification of tumors of the central nervous system: a summary. *Neuro Oncol*. 2021;23(8):1231-1251.

25. Saito T, Rehmsmeier M. The precision-recall plot is more informative than the ROC plot when evaluating binary classifiers on imbalanced datasets. *PLoS One*. 2015;10(3). doi:10.1371/journal.pone.0118432

26. Davatzikos C, Rathore S, Bakas S, et al. Cancer imaging phenomics toolkit: quantitative imaging analytics for precision diagnostics and predictive modeling of clinical outcome. *J Med Imaging*. 2018;5(01):1. doi:10.1117/1.jmi.5.1.011018

27. McNemar Q. Note on the sampling error of the difference between correlated proportions or percentages. *Psychometrika*. 1947;12(2):153-157. doi:10.1007/BF02295996

28. Leisenring W, Alonzo T, Pepe MS. Comparisons of predictive values of binary medical diagnostic tests for paired designs. *Biometrics*. 2000;56(2):345-351. doi:10.1111/j.0006-341X.2000.00345.x

29. DeLong ER, DeLong DM, Clarke-Pearson DL. Comparing the Areas under Two or More Correlated Receiver Operating Characteristic Curves: A Nonparametric Approach. *Biometrics*. 1988;44(3):837. doi:10.2307/2531595
